# Make it worth it: Effort-reward modulations on reinforcement-learning and prediction-error signaling across adolescence

**DOI:** 10.1016/j.dcn.2025.101559

**Published:** 2025-04-15

**Authors:** Anne-Wil Kramer, Lydia Krabbendam, Jessica V. Schaaf, Hilde M. Huizenga, Anna C.K. Van Duijvenvoorde

**Affiliations:** a Department of Developmental Psychology, University of Amsterdam, Amsterdam, the Netherlands; b Department of Developmental Psychology, Institute of Psychology, Leiden University, Leiden, the Netherlands; c Leiden Institute for Brain and Cognition, Leiden University, Leiden, the Netherlands; d Department of Clinical, Neuro, and Developmental Psychology, Vrije Universiteit Amsterdam, Amsterdam, the Netherlands; e Amsterdam Brain and Cognition Center, the Netherlands; f Medical Neuroscience Department, Donders Institute for Brain, Cognition, and Behaviour, Radboudumc, Nijmegen, The Netherlands

**Keywords:** Adolescent development, Reinforcement-learning, Effort-reward trade-off, Neuroeconomics, Dorsal anterior cingulate cortex, Prediction-errors

## Abstract

Adolescence is characterized by significant shifts in effort allocation. A well-known neuro-economic framework suggests that rewards help overcome potential effort costs. However, few studies have examined the neurobiological mechanisms by which rewards and associated effort costs drive adolescent learning. This study utilized functional magnetic resonance imaging in a sample of adolescents (*N* = 146, 13–25 years) and employed a reinforcement-learning paradigm that manipulated effort and reward levels, by varying task demands and varying potential rewards. The analysis of trial-by-trial learning signals (reward prediction errors) and behavioral learning performance demonstrated that greater reward levels enhanced adolescent learning, especially when faced with greater effort demands. Moreover, this effect was more pronounced in those experiencing greater effort demands: younger adolescents and adolescents who place less value on effort for demanding tasks. Neuroimaging results revealed that the dorsal anterior cingulate cortex (dACC) was a key region in signaling the interaction between reward and effort demands. That is, greater reward strengthened prediction error coding in the dACC, particularly under conditions of greater task demands, with these effects being more pronounced in younger adolescents and adolescents who place less value on effort for demanding tasks. These findings support a role for dACC in the engagement of cognitive control, especially in situations where more cognitive control would be beneficial despite its associated effort costs, such as in high-demanding learning situations. This comprehensive approach aims to inform strategies for supporting effort allocation in learning during this crucial developmental period.

## Highlights

• We examined the neurobiological mechanisms underlying how rewards and effort costs influence learning across adolescence.
• We observed that greater rewards enhanced learning performance across both low and high effort learning contexts.
• Younger adolescents and those valuing effort less learned more from high rewards, but only in high-effort learning contexts.
• Neuroimaging showed stronger prediction error coding for high vs. low rewards, but only in high-effort learning contexts.
• Dorsal ACC signals the interaction between effort and reward, and its activation shows age-related differences across adolescence.

## 1 Introduction

How do adolescents decide whether to invest cognitive effort? Prior research showed that adults base such a decision on a cost-benefit analysis where cognitive control is often seen as costly, deterring effort (Kurzban, 2016, Westbrook and Braver, 2015; Kool et al., 2017b, Kool et al., 2017a; Botvinick and Braver, 2015). However, rewards are known to increase the value of engaging cognitive control, a concept central to the Expected Value of Control (EVC) theory (Shenhav et al., 2013, Shenhav et al., 2016, Shenhav et al., 2017). Cognitive control refers to a set of higher-order cognitive functions, including inhibition, working memory and learning, that contribute to cognitive performance (Collins and Frank, 2013, Miller and Cohen, 2001, Yoo and Collins, 2022). Rewarding performance can improve cognitive control in adults on a variety of tasks (Boehler et al., 2014, Jimura et al., 2010; Locke and Braver, 2008; Leotti and Wager, 2010; Padmala and Pessoa, 2011; Schmidt et al., 2012). However, it is not always clear why and how rewards improve cognitive control performance. Here, we argue that rewards compensate for cognitive effort costs (why) by signaling that investing effort is worth the outcome (how), thereby mobilizing cognitive resources. This is particularly relevant to study in adolescence, a life phase known for high reward sensitivity and ongoing maturation of cognitive control.

Adolescent reward-sensitivity has been studied from a neurobiological perspective, in which prior studies indicate that the brain’s reward system responds more strongly to rewards in adolescents compared to children and adults (Braams et al., 2015, Silverman et al., 2015, Schreuders et al., 2018). High reward sensitivity can result in a tendency to gravitate towards rewards associated with risky behaviors, yet it can also encourage a focus on positive rewards, like enhanced motivation for academic achievement (Telzer, 2016, Van Duijvenvoorde et al., 2016). Alongside the maturation of the reward system, cognitive control undergoes age-related changes, gradually improving from childhood to late-adolescence (Crone and Dahl, 2012, Davidow et al., 2016, Satterthwaite et al., 2013), mimicking the maturation of prefrontal and parietal control regions. In the current study, we extend this typical maturational perspective on adolescent’s -effortful- cognitive control with a cost-benefit framework of cognitive control allocation during reward-based learning.

To effectively guide the allocation of cognitive control, one must possess the mature ability to differentiate between various levels of reward and effort demands, and to integrate reward value with these demand levels (Davidow et al., 2018). It seems, however, as if early-mid adolescents have not yet optimized this ability (Davidow et al., 2018). For example, prior research found that the ability to effectively use (high versus low) reward value to improve inhibitory control matures in late adolescence (age 19) (Insel et al., 2017, Insel et al., 2019). Results of these studies suggest that early-mid adolescents are not yet able to integrate reward value with cognitive control demands when they need to inhibit prepotent responses. Adolescence is a critical period for understanding the interplay between reward and cognitive control in learning environments. In contexts where more cognitively demanding strategies lead to better outcomes, Bolenz and Eppinger (2022) demonstrated that rewards enhance cognitive control. Adolescents increasingly adopted these demanding strategies when higher rewards were at stake, and this capacity improved throughout adolescence. However, a key question that remains is whether rewards can compensate for high effort demands that adolescents face in learning contexts.

Reinforcement learning (i.e., learning from rewards) is an ideal framework to assess this question as it allows for the manipulation of both reward value and effort demands in a controlled learning context. For instance, one may vary the amount of reinforcement that is at stake (as also done by Insel et al., 2019 and Bolenz and Eppinger, 2022) and the number of items that need to be learned in parallel, thereby increasing cognitive load (e.g., Master et al., 2020) and effort demands. This approach enables a deeper understanding of how adolescents balance effort and reward in complex learning environments. A recent behavioral reinforcement learning study in early-mid adolescents (age 13 – 15) that did vary effort demands, but not reward value, already provided support for reward as a compensatory mechanism for high experienced effort demands. Specifically, results showed that rewards (versus losses) improved learning performance, but only when effort demands were high (Kramer et al., 2023). Given adolescents’ high reward sensitivity, this characteristic could be strategically utilized in order to increase motivation to engage cognitive control in effortful learning contexts. Indeed, adolescents’ high reward sensitivity sometimes allows for greater cognitive flexibility (for a review see Van Duijvenvoorde et al., 2016) with studies observing better reward-based learning performance in adolescents compared to adults (Davidow et al., 2016; Eckstein et al., 2021) but see (Hauser et al., 2015). Building on these findings, the current study explored the dynamics between reward level and effort demands across adolescence, testing whether high rewards can offset effort demands in reinforcement learning. If so, high rewards (versus low) should be effective particularly when effort demands are high (versus low).

To clarify, we distinguish between two types of effort. The first is objective effort demands, which are experimentally manipulated by increasing task demands, such as the number of items to be learned simultaneously. As task demands increase, so does the required effort. The second is subjective value of effort, which reflects an individual’s perception of effort, is assessed by effort-discounting, and is often thought of as reflecting motivation to engage cognitive control (e.g., Westbrook et al., 2013; Westbrook and Braver, 2015; Kramer et al., 2021; Kramer et al., 2024). According to the value-based cognitive-control framework (Shenhav et al., 2013, Westbrook and Braver, 2015), subjective value of effort may increase when reward values increase, as higher rewards increase the perceived benefits, thereby shifting the cost-benefit balance.

Earlier studies have observed reward-based learning performance to increase with age across adolescence (Christakou et al., 2013, Master et al., 2020 Van der Schaaf et al., 2011), likely due to cognitive developmental differences (for reviews see Crone and Steinbeis, 2017; Luna, 2009; Satterthwaite et al., 2013). These cognitive developmental differences may also contribute to younger adolescents subjectively experiencing higher effort costs, as they need to draw on cognitive resources they may not easily access in order to achieve similar levels of performance as older adolescents. Besides age-related differences in the subjective experience of effort demands due to cognitive developmental differences, large individual differences (unaffected by age) in the subjective value (SV) of cognitive effort have earlier been observed in children (Chevalier, 2018), adolescents (Kramer et al., 2021) and adults (Westbrook et al., 2013, Westbrook et al., 2019), with lower SV of effort reflecting higher experienced task demands. Considering a cost-benefit framework of effort, this suggests that higher rewards are needed for those who experience higher effort demands. While this explains why rewards can improve performance, it does leave the question of what the neurocognitive mechanisms are.

A suggested idea is that rewards signal to the brain that more resources should be allocated to obtain something of value (e.g., Ballard et al., 2011; Krebs et al., 2011; Inzlicht et al., 2018; Nagase et al., 2018). A clear candidate that signals the requirement to allocate resources is the reward prediction error (PE) signal (Schultz, 2017, Schultz, 2016). The PE signal represents the difference between the expected value of a reward and the observed reward, is crucial for updating values associated with choices, and is therefore often referred to as the learning signal in RL frameworks (Schultz, 2016). PE signals are critical for adjusting cognitive and motivational resources in response to effort demands (Nagase et al., 2018). Particularly, a positive PE indicates that more value can be obtained than expected and may signal an important motivational request for allocation of more cognitive resources (Nagase et al., 2018).

In the current study, we used an RL context in a developmental population to examine how different effort demands and reward levels are associated with PE coding in reward-related brain regions, including the striatum and ventromedial prefrontal cortex (vmPFC) (Fouragnan et al., 2018), and the dorsal anterior cingulate cortex (dACC). While many roles have been assigned to dACC, one proposed function is the role of effort engagement (Touroutoglou et al., 2020). First, evidence for this can be found in the structure of dACC as a central communication hub that integrates information thereby motivating goal-directed action (Holroyd and Yeung, 2012, Shenhav et al., 2017, Touroutoglou et al., 2020). Indeed, dACC lesions have been found to lead to apathy and decreased motivation (Bonnelle et al., 2016). Functionally, greater dACC activation has been observed during cost-benefit weighing, and has been associated with greater willingness to exert effort (Bonnelle et al., 2016, Scholl et al., 2015, Scholl et al., 2017). In adults, dACC has earlier been found to be related to PE processing in effortful tasks specifically (Alexander and Brown, 2019, Vassena et al., 2017, Zendehrouh et al., 2014). Hence, variability in PE coding in dACC may relate to individual differences in responding towards effortful tasks. To summarize, in the current study we used functional magnetic resonance imaging (fMRI) to test whether and how rewards compensate for effort demands and benefit cognitive control allocation in adolescents. Given the varying results from previous studies on adolescents' ability to utilize reward to benefit cognitive control, this study includes a broad adolescent age range, from early adolescence to young adulthood (*N* = 146, 13 – 25 years). We examined the effect of reward (high > low) on (a) learning behavior and (b) PE signaling in key reward and effortful control regions, including the dACC, vmPFC, and striatum. Effort demands were manipulated by varying task demands in a reinforcement learning paradigm. We hypothesized that rewards enhance motivation to engage cognitive control, reflected in stronger PE signaling (i.e., higher beta estimates) and improved learning, particularly in high-effort tasks, consistent with the Expected Value of Control theory (Shenhav et al., 2013, Shenhav et al., 2016). We further hypothesized that the effects of reward on learning behavior and PE signaling would be particularly pronounced in:

1. Young adolescents, who experience higher cognitive effort demands due to ongoing maturation of cognitive control systems (Crone and Dahl, 2012, Satterthwaite et al., 2013, Master et al., 2020), and
2. Adolescents with low subjective value (SV) of effort, who may require higher rewards to offset perceived effort demands, as shown in prior work (Kramer et al., 2023).

While the effect of reward on PE-signaling is expected across the vmPFC and striatum (Schultz, 2016, Fouragnan et al., 2018), we expect that the reward x effort interaction is most prominently found in the dACC. The dACC plays a critical role in integrating cost-benefit information, effort engagement, and signaling the need for increased cognitive control (Shenhav et al., 2016, Alexander and Brown, 2019, Touroutoglou et al., 2020, Yee et al., 2021), particularly during high-effort tasks, where it is associated with both willingness to engage effort and PE processing (Bonnelle et al., 2016, Scholl et al., 2015). In addition, we explore whether age- and individual differences in subjective value of effort are present in these regions of interest. By elucidating how reward modulates PE-signaling, this study offers insights into the neural mechanisms linking motivation and cognitive control to learning across adolescence.

## 2 Method

### 2.1 Participants & procedure

A total of 156 participants aged between 13 and 25 years participated in this study. Participants were recruited through schools and online advertisements. We made careful efforts to ensure participants came from diverse educational backgrounds to promote generalizability and equity. That is, 39.4 % attended (pre-) vocational education, 32.5 % attended (pre-) higher education, and 28.1 % attended (pre-) university education. We also asked about the language participants spoke at home. Specifically, 72 % of participants spoke only Dutch at home, 25.2 % spoke Dutch and another language, and 2.8 % spoke only a non-Dutch language at home. No data on race, ethnicity, or socioeconomic status were collected.

One participant fell asleep and was therefore excluded from any analyses (N = 1). Based on frame-wise displacement values or signal-to-noise ratios, we excluded another six participants. Finally, three participants did not perform above chance level in any of the learning blocks and were therefore also excluded. The final sample thus consists of 146 participants, 77 female, M*age* = 19.71 years, SD*age* = 3.28 years; 13–25 years). The local review board approved this study (reference: 2021-DP-13298). All participants and parents of minors provided written informed consent. Participants were screened for MRI contraindications, reported no psychiatric or neurological disorders, and had normal or corrected-to-normal vision. Participants received payment for their participation (base rate of 37.50 euros) and could earn bonus money based on performance in the reinforcement-learning run (max bonus 12.50 euros).

Participants joined the laboratory for a 2.5 hour visit. They first performed two practice runs of the reinforcement learning run on a computer in a quiet room outside the MRI. They practiced a low effort – low reward run, after which they practiced a high effort – low reward run. After, participants performed four runs of the RL task in the scanner, and a structural T1 scan was made. After scanning, participants completed IQ measures in a quiet room with an experimenter, after which they performed a decision-making task and filled out questionnaires on a computer via Qualtrics (www.qualtrics.com).

### 2.2 Reinforcement learning task & design

To manipulate effort demands in an RL paradigm, we chose to manipulate the number of stimuli to be learned in parallel, a manipulation known to increase cognitive load in reinforcement learning (e.g., Collins and Frank, 2012; Collins et al., 2017; Master et al., 2020). Furthermore, a recent study found that adolescents discount reward value in RL tasks that differ in the number of stimuli (Kramer et al., 2023). This indicates that adolescents experienced learning four stimuli in parallel as more effortful than learning two stimuli in parallel.

Participants performed a two-choice probabilistic reinforcement learning task in the MRI scanner (see Fig. 2A). Participants were instructed to make a series of decisions between two pseudo words that were the same, except for one letter (i.e., homophones). They were instructed to learn which word in a pair was correctly spelled. A pseudo word is a word that does not exist but still follows logical grammar rules. We chose the stimuli to be pseudo words in order to examine acquisition of stimulus-outcome associations irrespective of pre-existing knowledge. We chose meaningful, language stimuli to relate to learning in typical adolescent learning environments. Each pseudo word-pair was accompanied by a picture of a common object (e.g. a chair) (e.g. Verburg et al., 2019). One of the two pseudo words within a word-pair was associated with a high probability of winning points, while the other pseudo word was associated with a low probability of winning points. The probabilities were 80 % and 20 % but were unknown to the participant. After each decision, participants were presented with the outcome, that is points, to enable them to learn the reward contingencies.

Participants performed the learning task in four different conditions varying in effort demands (low vs high) and reward level (low vs high). In low-effort runs, participants were learning two pairs of pseudo words in parallel, while in high-effort runs, participants were learning four pairs. In low-reward runs, participants could earn one point for correct choices and zero for incorrect choices, while in high-reward runs, participants could earn 10 points for correct choices and zero for incorrect choices. Participants were informed that their earned points would be converted into a monetary bonus they would receive at the end of the experiment (each point valued 0.10 euros) on top of their participation rate (37.50 euros).

Each run started with an instruction screen with text indicating whether the run would be low- or high-effort and whether they could earn low or high rewards. Each trial started with a fixation cross (jittered between 2000 and 4000 ms with steps of 500 ms). This was followed by the presentation of two pseudo words for 2000 ms during which participants were asked to select one of the stimuli. If no response was given within 2000 ms, the text ‘’too late’’ appeared on the screen. Missed trials were modeled separately in a nuisance regressor. These events happened rarely (1.1 % of trials). After participants made a choice, a selection frame appeared around the chosen option for 500 ms. Next, a fixation screen (again jittered between 2000 and 4000 ms) appeared, after which participants were presented with the outcome of their choice (+1 point or 0 points in the low-reward runs, and +10 points or 0 points in the high-reward runs). Whether the correct word appeared left or right on screen was counterbalanced across trials, and participants were instructed that the position of the words did not matter. Each word pair was presented 16 times. Thus, in low-effort runs, there were 32 trials (2 pairs x 16 times) as participants were learning two word pairs, and in high-effort runs, there were 64 trials (4 pairs x 16 times) as participants were learning four word pairs. As we performed separate analyses per effort-level, we decided to retain all trials in order to retain power and such that each word-pair was represented by an equal number of trials. Each run started with new pairs of pseudo words and accompanying pictures. Participants completed four separate fMRI runs with a short break in between runs. The order of the conditions was counterbalanced across runs and between participants.

### 2.3 Other measures

#### 2.3.1 Effort-discounting: subjective value of cognitive effort

Besides our objective task manipulation of effort demands (i.e., learn 2 words versus 4 words), we also assessed participants’ subjective value of effort (see Fig. 2 C) by using an effort-discounting procedure (see also Westbrook et al., 2013; Kramer et al., 2021). That is, participants were presented with choices between repeating a high-effort run (i.e. learn 4 words) for a large reward (i.e. 2 euros) or repeating a low-effort run (i.e. 2 words) for a smaller reward (starting at 1 euro). While the amount offered for the high-effort run remained fixed (i.e. 2 euros), the reward offered for the low-effort run varied until the participant is indifferent between the two effort-reward combinations. That is, when participants chose the high-effort option, the reward offered for the low-effort option increased on the next trial but decreased otherwise. This in- or decrease is always half as much as on the previous trial. After six trials, the amount offered for the low- effort option is taken as the indifference point, representing a participants’ subjective value (SV) of cognitive effort. A higher SV, indicates a higher willingness to invest effort. We found SV of effort to be unrelated to age (Pearson’s *r* = -.08). First, we analyzed the effect of SV as a continuous measure. Subsequently, given our hypothesis and for ease of interpretation and visualization purposes, in the follow-up analyses, we split our sample into two groups by means of a median split: low versus high SV of cognitive effort.

#### 2.3.2 Self-report ratings of effort and motivation

To assess participant’s subjective experience of the different RL runs in terms of effort and motivation, we asked them to rate each of the four RL runs on both perceived effort and motivation using a 5-point Likert scale after completing each run (so four times in total). Specifically, we asked ‘Think back to how much effort it cost you to learn during this block of the learning task. Choose the option that fits best. The task cost me…’. Participants answered on a 5-point Likert scale, ranging from 1 = no effort at all, to 5 = a lot of effort. In addition, we asked ‘Think back to your motivation during this block of the learning task. Choose the option that best matches your motivation during the task. During the task, I felt…’ Participants answered on a 5-point Likert scale, ranging from 1 = not motivated at all, to 5 = very motivated.

#### 2.3.3 IQ measures

To assess cognitive ability, participants performed the matrix reasoning, similarities and digit span subtests of the WISC or WAIS (depending on age). We used a standardized normed sum score of these tests as covariate in our analyses.

### 2.4 Behavioral analyses

*Generalized linear mixed model.* To analyze learning (choice behavior in the RL task), and to test our hypotheses that reward benefits learning 1) particularly when effort demands are high, and 2) particularly for young adolescents, we fitted a logistic generalized linear mixed model to decisions (correct coded as 1, incorrect as 0). Our model included fixed effects of effort, reward, linear age, linear trial, and all interactions.

Next, to test the hypothesis whether rewards 3) improve learning particularly for adolescents with low subjective value (SV) of cognitive effort, we first fitted a logistic generalized linear mixed model including fixed effects of trial, effort, reward, age and subjective value, including all interactions. To follow-up interactions and isolate the effects of reward across specific effort levels and subjective value (SV) groups we tested the main effect of reward in four separate models: for each effort level (low and high) and SV group (low and high). In addition, as a sensitivity analysis, we also ran our main model on learning accuracy with IQ and sex as covariates. This did not alter the results (see SOM Table S6). In all models, we standardized continuous independent variables (i.e., age and trial), and we contrasted categorical variables (i.e. reward and SV group: low = −1, high = 1). Additionally, in all models, we included a random intercept per participant. For these analyses, we used the lme4 package (Bates et al., 2014) in R version 4.1.3 (R Core Team, 2023).

Finally, we applied a Bonferroni correction to account for multiple comparisons in all follow-up analyses. Specifically, p-values were multiplied by 2 for analyses conducted per effort level and by 4 for analyses conducted per effort level and SV group. Uncorrected p-values are reported, with results failing to survive Bonferroni correction explicitly noted in the manuscript.

### 2.5 Computational modeling of behavioral data

We modeled choices with an extended Rescorla-Wagner reinforcement learning model (Wagner and Rescorla, 1972; Sutton and Barto, 2018). In this model, participants learn, that is, update the expected value of stimuli (i.e., spellings of pseudo words) through prediction errors, that is, differences between observed outcomes (i.e., points) and previous expected values of chosen stimuli. At the start of the experiment, the expected value of the correct and incorrect pseudoword spelling is equal. The model contains two parameters: the learning rate, that captures how fast participants update expected values based on prediction errors, and the inverse temperature, that captures to what extent participants use expected values to guide choices. To test for effects of the independent variables effort, reward, and age, we simultaneously (i.e., within the computational model) regressed model parameters on these independent variables. We compared model fit between a model including all three effects and a model with effort and reward effects but without age. A model comparison method showed that the model without age described the data best. From this best fitting model, the estimated prediction errors (PE; positive and negative) and expected value estimates were subsequently used as unstandardized trial-level parametric regressors in the fMRI analyses, modulating the impulse function at the moment of feedback onset (positive prediction error, negative prediction error), and at the moment of choice (expected value). As we were mainly interested in prediction error-related neural activity, we focus on the prediction error results. The regressors were mean-centered (not standardized) per participant per regressor per condition. Full model specification is reported in the SOM (section b).

### 2.6 fMRI acquisition

We used a 3 T Philips scanner (Philips Achieva) with a 32-channel head coil. Before the reinforcement learning run, a high-resolution T1 scan was obtained for anatomical reference (TR = 8.33 ms, TE = 3.84 ms, voxel size = 1 × 1 × 1 mm, field of view (FOV) = 240 × 220 mm). The reinforcement learning run was projected on a screen that participants viewed through a mirror on the head coil. Functional scans were acquired during three runs of 290 dynamics each, using T2 * echo-planar imaging (EPI), with TR = 2.0 s, TE = 2.8 ms, 36 slices, voxel size = 3 × 3 × 3 mm; field of view (FOV) = 240 × 220 mm. The first three volumes were discarded to allow for equilibration of T1 saturations effects. To correct for B_0_ inhomogeneities, all functional scans were followed by a pepolar fieldmap scan (a.k.a. ‘’topup’’) in the opposite direction of the EPI scan (i.e. PA).

#### 2.6.1 fMRI preprocessing

Data preprocessing was performed using fMRIprep with the HALFpipe pipeline (Waller et al., 2022). Motion correction was conducted using ICA-AROMA, with noise components identified and regressed out. Spatial smoothing was applied with a 6 mm full-width at half-maximum (FWHM) Gaussian kernel. Temporal filtering was performed using a Gaussian high-pass filter with a cutoff of 100 seconds. Functional images were coregistered to each participant’s T1-weighted anatomical image and normalized to MNI152 space using nonlinear transformations. Slice timing correction was applied based on the recorded acquisition order. Skull stripping was conducted using FSL BET. The data were acquired with a voxel size of 3.0 × 3.0 × 3.0 mm and resampled to 2 mm isotropic resolution after normalization. Physiological and motion-related confounds were controlled by regressing out six motion parameters (three translational, three rotational) along with their first-order derivatives. Four participants exceeded an FWD threshold of 0.90 mm in two conditions and were excluded from further analyses. The remaining participants exhibited minimal head motion (Mean = 0.16 mm, SD = 0.11 mm). In addition to automated quality control, visual inspection of signal-to-noise ratio (SNR) was performed, leading to the exclusion of two participants due to poor data quality.

#### 2.6.2 fMRI general linear model

We used the general linear model (GLM) in FSL version 6.0 to perform statistical analyses on individual subjects’ fMRI data. The GLM that was used for modeling the brain response in each run held 6 predictors with a zero duration and their derivatives: 4 predictors that modeled the brain response at the moment of outcome onset: (1) a modulated predictor (weighted by PE values) for negative PEs, (2) an unmodulated predictor (weighted as 1) for negative PEs, (3) a modulated predictor (weighted by PE values) for positive PEs, (4) an unmodulated predictor (weighted as 1) for positive PEs, and 2 predictors that modeled the brain response at the moment of stimulus onset: (5) a modulated predictor (weighted by expected values) for expected value, and (6) an unmodulated predictor (weighted as 1) for expected value. The PE regressors were neither orthogonalized nor standardized (Schaaf et al., 2024) but were mean centered per participant per regressor per condition. All predictors, including the PE Predictor, were paired with a matching temporal derivative. As positive and negative PEs reflect different processes, it is common to model them separately (e.g., Fouragnan et al., 2018; Nagase et al., 2018). We did not contrast them. We modeled missed trials as zero events. All regressors were convolved with the hemodynamic response function (HRF). Runs per participants (i.e., four runs per participant) were then combined with a fixed-effects analysis. For the whole-brain analyses, these subject-level maps were submitted to a random-effects group-level analysis (Flame 1 +2). In this study, our primary interest encompassed both positive and negative prediction error (PE) coding. However, due to the infrequent occurrence of negative PEs, our analyses focused on the neural coding of positive PEs. The rarity of negative PEs can be attributed to participants’ learning over time and the task design, wherein feedback was provided with an 80 % probability. We therefore analyzed the key contrast that focused on the positive PE. We first present results across effort conditions. We next present results per effort condition separately. That is, we report effects of reward (high > low) and interactions between reward and age in both low-effort and high-effort runs.

#### 2.6.3 ROI selection and fMRI analyses

**Fig. 1:**
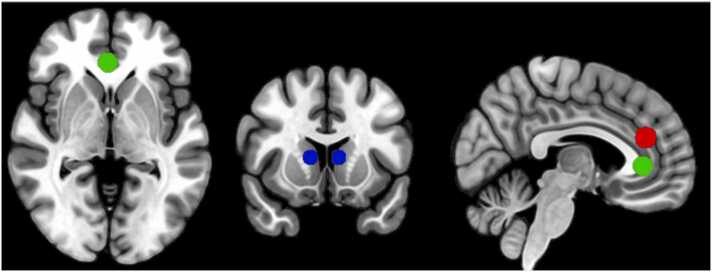
Regions of interest: red depicts dorsal ACC, green depicts vmPFC and blue depicts dorsal striatum.

Based on a meta-analysis on PE representation (Fouragnan et al., 2018) and literature on PE representation in adolescents specifically (e.g. Cao et al., 2019) we included ROIs from dACC [4 36 20], vmPFC [0 34 0], and dorsal striatum [right: 10 14–6; left: −10 4–6]. We drew 8 mm spheres around those coordinates (Fig. 1). We used FSL (fslmaths) to perform the ROI analyses. We extracted PE-related parameter estimates per subject for each of the four effort-reward runs. Higher parameter (i.e., beta) estimates represent stronger PE coding. We then performed repeated-measures (RM)-ANOVAs on the ROI values with effort and reward as within factor and age as continuous predictor, including all interactions. Next, we performed a similar RM-ANOVA and added SV as a continuous measure. To test the effects of reward across specific effort levels and subjective value (SV) groups we next tested the main effect of reward in four separate models: for each effort level (low and high) and SV group (low and high), again with reward as within factor and including age as continuous covariate. Finally, we applied a Bonferroni correction to account for multiple comparisons in all follow-up analyses. Specifically, p-values were multiplied by 2 for analyses conducted per effort level and by 4 for analyses conducted per effort level and SV group. Uncorrected p-values are reported, with results failing to survive Bonferroni correction explicitly noted in the manuscript.

## 3 Results

### 3.1 Subjective ratings of effort and motivation

We first examined whether the subjective experience of the different task runs followed our effort and reward manipulation by examining self-report ratings of effort and motivation. We only report significant results. Full model output can be found in the SOM (Table S4). A linear mixed model with effort and reward as within-variables and age as additional predictor on self-reported effort showed that high-effort runs were experienced as more effortful than low-effort runs (main effect effort: β = 0.47, p < .001). We thus conclude that the subjective experience of the different task runs followed our effort and reward manipulations. As an aside, we also observed a reward * age interaction such that low-reward tasks were experienced as more effortful for younger ages (β = 0.06, p = .04).

A similar linear mixed model on self-reported motivation with effort and reward as within-variables and age as additional predictor showed that runs with high rewards were experienced as more motivating than runs with low rewards (main effect reward: β = 0.26, p < .001). We also observed a reward * age interaction such that low-reward tasks were experienced as less motivating for younger ages (β = −0.05, *p* = .02). Together these findings show that younger adolescents experienced low-reward tasks as more effortful and less motivating than older adolescents.

### 3.2 Learning behavior

**Fig. 2:**
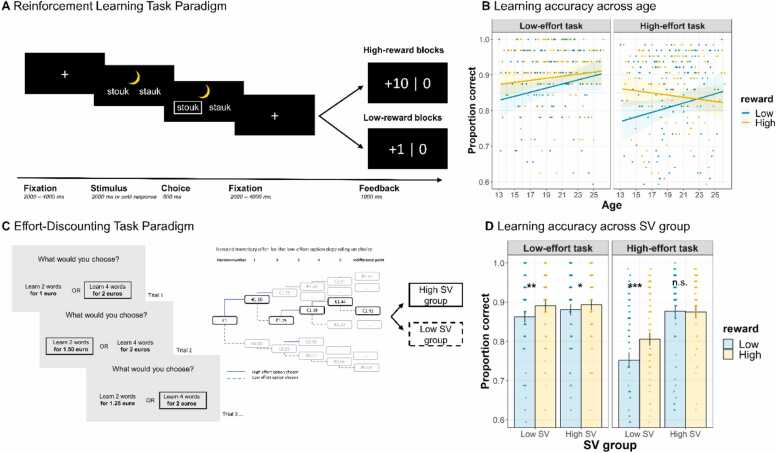
(A) Reinforcement learning task paradigm (B) Learning accuracy per effort level as a function of reward across age (C) Effort-discounting task paradigm, and (D) Learning accuracy per effort level as a function of reward and SV group. Note: in panel B, shaded areas represent 95 % CI. In panel D, error bars denote + /- 3 SEM. In panel D, the effect of reward on accuracy in the low-effort task in the high SV group is not significant after Bonferroni correction.

First, we used generalized linear mixed models on trial-level learning performance per effort level, to examine our hypotheses that reward benefits learning 1) particularly when effort demands are high, and 2) particularly for young adolescents. Behavioral results are depicted in Fig. 2B. We only report significant results. Full model output is included in the SOM (Table S5).

First, we observed a significant effort * reward * age interaction (β = −0.04, *p* = .04). Follow-up analyses per effort-level including reward * age interaction (see SOM Table S5) revealed that reward affected learning accuracy in both low-effort (β = 0.15, *p* < .001) and high-effort (β = 0.13, *p* < .001) runs, in that high rewards resulted in higher accuracy (see SOM Table S2 for means). However, only in high-effort runs we observed an interaction between reward and age (high effort run: β = −0.12, *p* < .001; see Fig. 2B; low-effort run *p* = .79. Follow-up analyses using logistic linear mixed models in the high-effort runs within each age group and including a main-effect of reward, showed that reward particularly benefited high-effort learning performance in younger adolescents, with reward effects decreasing with increasing age: 13 – 15 year olds (β = 0.24, *p* < .001), 16 – 18 year olds (β = 0.19, *p* < .001), 19 – 21 year olds (β = 0.15, *p* = .001) and even slightly hampering performance in 22 – 25 year olds (β = −0.08, *p* = .04; not significant after Bonferroni correction; *p* = .16).

Second, we used a similar generalized linear mixed model on trial-level learning performance, to examine our hypothesis 3) that reward benefits learning particularly for adolescents with low SV of effort (controlling for age). The full model-output is included in the SOM (Table S9). Results are depicted in Fig. 2D. First, we observed a main effect of subjective value (β = 0.29, *p* < .001), and interactions between effort and subjective value (β = 0.18, *p* < .001), and between reward and subjective value (β = −0.07, *p* < .001). For ease of interpretation and visualization purposes, follow-up analyses were done per effort level (low and high) per SV group (low and high SV). Full model output is included in the SOM (Table S10).

In the low SV group, reward affected learning accuracy in both low-effort (β = 0.14, *p* < .001) and high-effort runs (β = 0.19, *p* < .001), in that high rewards resulted in higher accuracy. However, in the high SV group, reward only improved learning accuracy in low-effort runs (β = 0.12, *p* = .03; not significant after Bonferroni correction, *p* = .12, see SOM Table S11), and not in high-effort runs (β = −0.02, *p* = .64). Together, behavioral results do not support the hypothesis that 1) reward benefits learning particularly when effort demands are high: they do so for both. Yet, results do support the hypotheses that reward improves learning particularly for those experiencing high effort demands: 2) young adolescents (though only when effort demands are high), and 3) adolescents with low SV of effort.

### 3.3 Prediction error coding

**Fig. 3:**
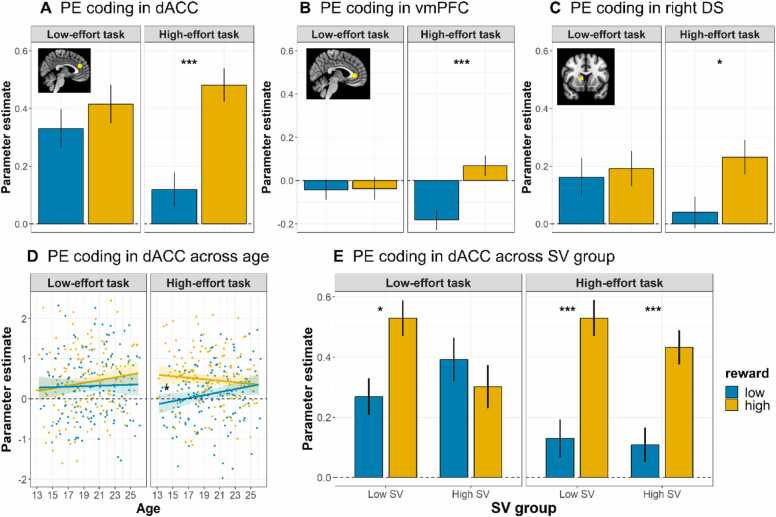
Strength of PE-related activations in (A) dACC, (B) vmPFC, and (C) right dorsal striatum. Strength of PE-related activations in dACC (D) across age, and (E) across SV group. Note: *p < .05, * *p < .01, * **p < .001.

We next examined whether reward strengthened positive PE coding 1) particularly in high-effort tasks, 2) particularly for young adolescents, and 3) particularly for adolescents with low SV of effort. We tested this in each of our four ROIs. We only report significant effects on associated positive PE activations. Full model output is included in the SOM (Table S9). Results are depicted in Fig. 3.

To test our first two hypotheses - that reward strengthened positive PE coding 1) particularly in high-effort tasks, and 2) particularly for young adolescents - results showed a main effect of reward on PE coding across effort levels in dACC (*F*(1, 576) = 13.23, *p* < .001) and vmPFC (*F*(1, 576) = 7.24, *p* < .001) but not in right or left striatum. We found no main effects of effort in any of our ROIs. We observed a main effect of age in the right striatum (*F*(1, 576) = 4.07, *p* = .03), but not in the left striatum, dACC or vmPFC (see SOM Table S7).

Next, we found reward * effort interactions in dACC (*F*(1, 576) = 3.97, *p* = .04) and vmPFC (*F*(1, 576) = 8.14, *p* < .001), and a reward * effort * age interaction in dACC, (*F*(1, 576) = 4.77, *p* = .02) (see SOM Table S7).

To unpack these interactions, we performed follow-up RM-ANOVAs testing reward * age effects for the dACC per effort level. We observed a main effect of reward only in the high effort task *(F*(1, 288) = 18.30, *p* < .001), and a reward * age interaction in the dACC only in the high effort task *(F*(1, 288) = 4.88, *p* = .03; see Fig. 3D and SOM Table S8). Follow-up tests per age group showed that the effect of reward (high > low) in the high-effort task was strongest in younger age groups and decreased with age: 13 – 15 year olds (β = 0.40, *p* = .001), 16 – 18 year olds (β = 0.20, *p* = .01), 19 – 21 year olds (β = 0.16, *p* = .06) and 22 – 25 year olds (β = 0.09, *p* = .27).

Results from follow-up RM-ANOVA’s in our other ROIs showed a main effect of reward on PE coding only in high-effort tasks in vmPFC (Fig. 3B), *(F*(1, 288) = 16.13, *p* < .001), and right striatum (Fig. 3C and SOM Table S8) *(F*(1, 288) = 7.12, *p* = .01), but not in left striatum (*p* = .60). In all regions high compared to low reward resulted in greater PE activation.

Finally, to test our third hypothesis - that reward strengthened positive PE coding particularly for adolescents with low SV of effort, we first performed a RM ANOVA with SV (continuous) x effort x reward, controlling for age. These results showed no significant effects of continuous SV in any of our ROIs. To keep analyses consistent, and given our hypothesis, RM-ANOVA’s per SV group (low and high) revealed in both SV groups only significant effects of reward on PE coding in dACC. Results showed, for the low SV group, main effects of reward on PE coding in both low-effort (*F*(1, 140) = 5.11, *p* = .03; not significant after Bonferroni correction, *p* = .12, see SOM Table S12) and high-effort tasks *(F*(1, 140) = 11.35, *p* < .001) (Fig. 3E). However, for the high SV group, main effects of reward on PE coding were found only in the high-effort tasks *(F*(1, 140) = 8.20, *p* < .001) and not in the low-effort tasks (*p = .57*). Thus, for the high SV group, In dACC, high compared to low reward resulted in greater PE activations.

Together, results show that PEs were coded stronger in high compared to low reward conditions, and that this effect was present only in high, and not in low effort conditions (supporting hypothesis 1). dACC showed age-related differences, demonstrating that, when effort costs are high, providing high rewards resulted in stronger PE coding in dACC particularly for young adolescents (supporting hypothesis 2). Finally, dACC also showed SV group differences, such that high rewards strengthened PE coding in both low- and high-effort conditions for adolescents with low SV of effort but not for adolescents with high SV of effort (supporting hypothesis 3).

### 3.4 Whole brain results

**Fig. 4:**
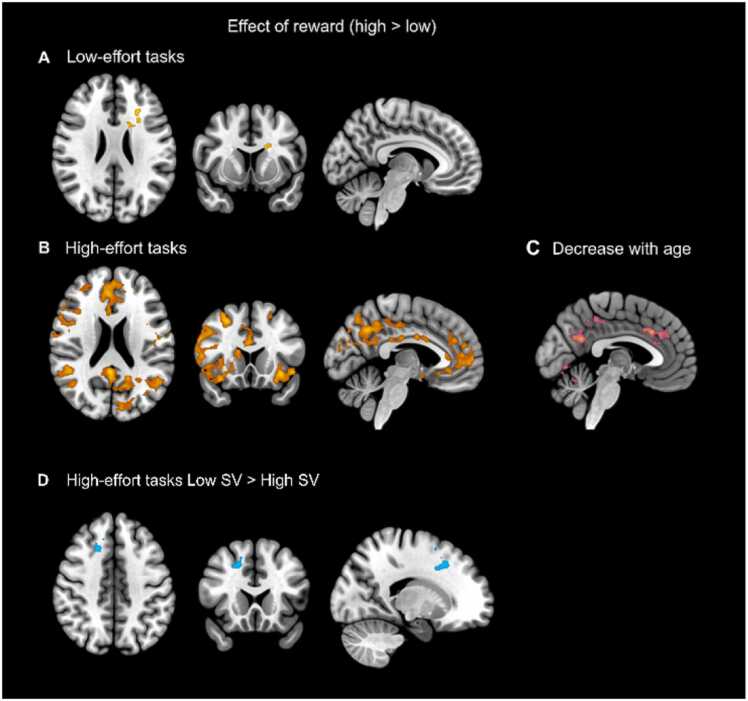
Whole brain positive PE-activations for the effect of reward (high > low) in (A) low-effort tasks and (B) high-effort tasks. A negative age effect that was present only in (C) high-effort tasks, and a difference in the effect of reward on positive PE-activations in (D) high-effort tasks between the low and high SV group. *Note*: A cluster-wise multiple comparison correction was used with *z*-threshold 2.3.

To ensure our ROI approach did not restrict the understanding of our findings we included whole brain analyses for effects of reward and age on positive PE coding in each effort condition separately. In low effort conditions, we found effects of reward (high>low) on positive PE-activation in middle frontal gyrus and caudate only, while in high effort conditions, we found widespread effects of reward on positive PE-activation in (dorsal anterior) cingulate cortex, frontal regions, striatal regions and precuneus cortex (see Fig. 4B). In addition, in high-effort runs, we found effects of reward (high>low) on positive PE-activations that were stronger for younger ages in (dorsal anterior) cingulate cortex, precuneus cortex and other regions (see Fig. 4C). Finally, in high-effort runs, we found effects of reward (high>low) on positive PE-activation that were stronger for adolescents with low compared to high SV of effort in supplemental motor area and mid anterior cingulate cortex (Fig. 4D). All activations are reported in SOM Tabel S13.

## 4 Discussion

In the current study we aimed to examine neurobiological mechanisms of how rewards and associated effort demands drive adolescent learning. Adolescents are often motivated by rewards, whether academic or social, making it critical to understand how rewards shape learning and enhance motivation. For example, a student may work harder for a challenging exam if a high grade leads to a scholarship, illustrating how anticipated rewards influence effort. Adolescents frequently face tasks requiring cognitive effort for subjects they may not value (yet), raising the question of whether performance-based rewards can address motivation issues in high-effort tasks. This approach builds on prior findings that highlight specific brain regions' roles in processing rewards and effort (e.g., Westbrook et al., 2019; Yee et al., 2021). By studying the neural mechanisms, we can better understand individual variations in responses to rewards and effort demands, and understand how the involved brain regions differ across adolescence. We therefore used fMRI to test whether and how rewards compensate for effort demands in a reinforcement learning framework. We did so in a sample of 146 adolescents between ages 13–25 years. We tested the effect of reward (high > low) on a) learning behavior and b) PE coding in key reward and effort-related regions including the dACC, vmPFC, and striatum. We hypothesized that rewards compensate for effort costs and are beneficial to engage cognitive control 1) particularly in high effort conditions and particularly for those experiencing high effort costs, that is, 2) young adolescents and 3) adolescents with low subjective value of effort. Our discussion is organized alongside the lines of our hypotheses.

In contrast to our first hypothesis, behavioral findings indicated that high rewards enhanced learning performance in both low and high effort tasks. Neuroimaging findings, however, showed that reward strengthened PE coding in dACC, striatum and vmPFC in high effort tasks only. Further, whole brain analyses revealed widespread PE activation in response to high versus low rewards, only in high effort tasks. Together, these results align with the computational framework of an effort-reward trade-off in effortful decision making and learning (Shenhav et al., 2013, Shenhav et al., 2016). These findings provide insights into how adolescents weigh effort against reward in the context of reinforcement learning. Particularly, they show that adolescents strategically use reward value relative to effort demands to benefit their learning performance. In addition, results point to PE signaling in striatum, vmPFC and dACC as a potential neural mechanisms by which reward translates into better learning performance. These results further support the role of the dACC as reward-effort integration mechanism (Shenhav et al., 2016; Yee et al., 2018; Yee et al., 2021) in adolescence. While one earlier study has demonstrated the role of dACC in encoding subjective motivational value to modulate behavioral performance in a task with high effort demands in adults (Yee et al., 2021), ours is the first study to show that this is also true for adolescents in a learning context.

Next, we obtained evidence for our second hypothesis – that in high effort conditions, high versus low rewards would be especially beneficial for younger adolescents. This second hypothesis was supported, as we only observed reward by age effects in high but not low effort conditions. Particularly these results showed that early-mid adolescents are able to strategically let reward value guide their cognitive control allocation in reinforcement learning. This result is consistent with the reinforcement learning study of Kramer et al. (2023) – where reward aided learning in high-effort conditions only –, but not with the inhibition study of Insel et al. (2017), who found that the ability to let value guide cognitive control allocation matures only in late adolescence. Potentially, this discrepancy with prior results (Insel et al., 2017) may stem from the higher cognitive control demands of inhibition as compared to our effort demands manipulation in the reinforcement learning task. This explanation is supported by the observation that in the paradigm of Insel et al. (2017) performance increased with age in both low and high reward conditions, whereas our findings suggest that early adolescents can match older peers’ performance levels in high reward conditions only. Further studies should investigate in which task contexts rewards may be most beneficial to upregulate adolescents’ cognitive performance.

These behavioral findings were, furthermore, mirrored by neuroimaging findings. Consistent with previous research on PE signaling (Fouragnan et al., 2018), the vmPFC and striatum exhibited increased PE activation with higher reward levels. However, they showed limited sensitivity to variations in effort demands or age. In contrast, PE activation in the dACC demonstrated sensitivity to interactions among reward, effort, and age. That is, the impact of reward on PE coding was greater for young adolescents in high but not low effort conditions. This aligns with earlier research in adults showing that dACC plays a key role in merging motivational value with cognitive demands and translating this into goal-directed behavior (Touroutoglou et al., 2020; Shenhav et al., 2013, Shenhav et al., 2016; Yee et al., 2021). Our findings thus suggest that also in early to mid-adolescents cost-benefit tradeoffs are related to dACC signaling. Results also suggest that by increasing rewards, adolescent’s reward sensitivity (for reviews see Crone and Dahl, 2012; Davidow et al., 2018; Telzer, 2016; Van Duijvenvoorde et al., 2016) can be utilized to offset high perceived effort costs and boost reinforcement learning.

Finally, our behavioral findings corroborate our third hypothesis – that adolescents with low but not high subjective value of effort require greater rewards to enhance performance particularly in high effort conditions. That is, our results showed that adolescents who placed low value on effort required greater rewards to enhance performance in both low and high effort conditions, while adolescents who placed high value on effort did not benefit from greater rewards in either low- or high effort conditions.

Neuroimaging findings mirrored behavioral results for the low SV group, showing enhanced signaling of PE’s in response to high versus low reward in dACC for adolescents with a low subjective value of effort in both low and high effort conditions, while adolescents with a high subjective value of effort only showed this effect of reward on PE coding in the high effort condition, further aligning with the view of dACC as reward-effort integration mechanism (Shenhav et al., 2016, Yee et al., 2021).

These results show that individuals with a lower subjective value of effort may particularly benefit from high versus low reward. Future longitudinal research should investigate whether it is beneficial to reward these adolescents to eventually increase their (intrinsic) valuation of effort, potentially leading them to engage in more effortful learning endeavors, as recent research has also demonstrated that providing immediate external rewards for performance could enhance intrinsic motivation (Liu et al., 2022).

By integrating principles of reinforcement learning with neuroeconomic approaches to value-based decision-making, we show that how adolescents value reward and how they value effort modulates their learning, and demonstrate that these functions operate within a common computational framework. By varying effort demands with task demands, and examining individual differences in age and subjective value – we obtained strong support that providing high versus low rewards offsets effort demands in reinforcement learning. This aligns well with the mechanistic expected value of control (EVC) framework, in which dACC plays a central role. This framework states that dACC integrates information about the expected value of control, the amount of control that must be invested, and the (subjective) cognitive effort demands (Shenhav et al., 2013). Our findings that dACC activation in adolescents correlates with PEs, a signal that represents the difference between the expected and observed value of a reward, and that this correlation varies depending on effort demands of the context, support this framework. As such, our study contributes to developing a broader mechanistic understanding of how dACC integrates value during motivated cognitive control across adolescence: specifically tailored to effort demands of the context.

A strength of the current study is that we were able to use a decision-making (i.e., effort-discounting) task to assess individual’s *subjective* value of effort in an associated decision task. This way we could assess the inherently implicit subjective value of effort in a relatively objective manner, as opposed to self-report which relies on the assumption that behavioral motives are consciously accessible and declarative, while motivation and decisions to invest cognitive effort often arise from inaccessible and non-declarative cognitions and feelings (Fulmer and Frijters, 2009). By linking subjective value of effort to neural PE signaling, our results show that PE signals in the brain differ depending on subjective effort valuation (independent of age), and thus that how someone values effort affects learning processes. Moreover, our study's inclusion of an adolescent sample from varied educational backgrounds enhances the generalizability of our findings on PE signaling and cognitive control.

The current study also knows some limitations. First, although we measured subjective value of effort with an effort-discounting task, inferring motivational value in a data-driven manner, this was only done after task completion. To obtain a better understanding of the temporal dynamics of effects of motivational value on effort investment during learning, future research could include intermittent assessments of the subjective value of effort. Furthermore, although we included a broad age-range, our study’s cross-sectional design limits our ability to observe how the relationship between reward, effort, PE signaling and learning changes with age. For example, future studies should investigate whether, after stopping to provide rewards, those effects are sustained over time, by using longitudinal designs. If so, this would provide further evidence for the idea that intrinsic motivation may be triggered by providing external rewards (Liu et al., 2022; Deci, 1971).

Another potential limitation is the way in which the computational model is formulated. For example, we did not include a working memory component. Models including such a working memory component have sometimes been shown to better describe data than models excluding such a component in similar reinforcement learning tasks (see e.g., Collins and Frank, 2012). In adolescents (Master et al., 2020) it has earlier been found that older adolescents tend to use more working memory related processes than younger ones. Future studies may add a working memory component to the computational model to investigate whether working memory use also changes as a function of effort and reward.

Finally, a limitation of this study is the ecological validity of the experimental task. While computerized tasks provide precise control over variables, they may not fully capture the complexity of real-world learning environments. Although we included stimuli which arguably better match learning contexts than stimuli typically encountered in reinforcement learning tasks, generalizability to real-world learning settings remains an open question (Yarkoni, 2022, Holleman et al., 2020). Future research could address this by incorporating real-world classroom studies or longitudinal designs to examine how adolescents apply effort-reward trade-offs in everyday learning. While our task isolates key cognitive and motivational mechanisms, complementing such approaches with naturalistic studies will help clarify their broader applicability.

These findings may have broader implications for educational practices and interventions aimed at enhancing learning and motivation among adolescents. They suggest that reward structures in learning environments need to be tailored to the cognitive demands of the task to maximize their motivational impact and stimulate cognitive control allocation. When tasks are perceived as effortful, the strategic use of rewards could enhance learning engagement and learning outcomes. Furthermore, our results point towards the positive side of early-mid adolescent’s reward sensitivity, and support the view that high reward sensitivity may be leveraged to play an adaptive role by encouraging effort investment (Telzer, 2016, Van Duijvenvoorde et al., 2016).

In addition, current theories of reinforcement learning often do not account for the role of effort in learning (Jarvis et al., 2022), yet our results suggest that they should as the brain signals PEs differently depending on effort demands of the context and effort valuation of the individual. Finally, it is important to note that effort can also be valuable and feel rewarding in its own right (Inzlicht et al., 2018). Yet, here we show that this is subject to individual differences, and that the subjective value of effort can be reflected in individual’s choice behavior when confronted with high and low effort options.

While this study examines effort and motivation in the context of learning, similar mechanisms apply across other domains. Effort discounting research suggests that individuals weigh effort costs differently depending on the type of reward, with variations observed in monetary, social, health-related, and environmental decision-making (Seaman et al., 2016, Vlasceanu et al., 2024). These findings highlight the broader relevance of effort-based choices beyond academic settings. Incorporating insights from other domains could further refine our understanding of how adolescents allocate effort across different contexts.

## 5 Conclusion

In conclusion, by combining a reinforcement learning framework with a neuro-economical perspective on effort and reward, we obtained behavioral and neural evidence that effects of reward on learning vary depending on the context’s effort demands, adolescents’ age and adolescents’ subjective value of effort. Our results contribute to the understanding of how adolescents’ reward sensitivity can be leveraged to promote effort investment. Furthermore, they highlight the importance of taking adolescent’s individual differences in effort valuation into account.

As adolescents often find themselves in situations in which they are required to perform effortful learning tasks that they do not intrinsically value (yet), thereby leading to low motivation to invest cognitive effort, the results of the current study suggest that rewarding performance in high effort situations may make it worth their while. This knowledge could be used in educational settings, suggesting that, especially early-mid adolescents may benefit from environments in which high-effort tasks may be explicitly better rewarded than low-effort tasks to enhance motivation and effort investment, and improve learning. Furthermore, the observed individual differences in subjective value of effort underscore the importance of personalized approaches to motivate adolescents to invest effort in learning.

## Funding

AK was supported by the Start Impulse grant to NeuroLabNL from the Dutch National Science Agenda (NWA) [grant number 400.17.602]. LK has received funding from the European Research Council (ERC) under the European Union's Horizon 2020 research and innovation programme (grant agreement n° 648082). HH and JS were supported by a VICI grant awarded by the Netherlands Organization of Scientific Research (NWO) [grant number 453-12-005]. AvD was supported by an ORA-grant [grant number 464-15-176] (partly) financed by the Dutch Research Council (NWO) and by the Social Resilience and Security program (Leiden University). The funding source had no role in the study design, collection, analysis or interpretation of the data, writing the manuscript, nor the decision to submit the paper for publication.

## CRediT authorship contribution statement

**Anne-Wil Kramer:** Writing – original draft, Visualization, Validation, Software, Project administration, Methodology, Investigation, Formal analysis, Data curation, Conceptualization. **Lydia Krabbendam:** Writing – review & editing, Supervision, Funding acquisition, Conceptualization. **Jessica V. Schaaf:** Writing – review & editing, Formal analysis. **Hilde M. Huizenga:** Writing – review & editing, Supervision, Methodology, Funding acquisition, Conceptualization. **Anna C. K. van Duijvenvoorde:** Writing – review & editing, Supervision, Methodology, Funding acquisition, Conceptualization.

## Declaration of Competing Interest

The authors declare that they have no known competing financial interests or personal relationships that could have appeared to influence the work reported in this paper.

## References

[bib1] Alexander W.H., Brown J.W. (2019). The role of the anterior cingulate cortex in prediction error and signaling surprise. Top. Cogn. Sci..

[bib2] Ballard I.C., Murty V.P., Carter R.M., MacInnes J.J., Huettel S.A., Adcock R.A. (2011). Dorsolateral prefrontal cortex drives mesolimbic dopaminergic regions to initiate motivated behavior. J. Neurosci..

[bib3] Bates, D., Mächler, M., Bolker, B., & Walker, S. (2014). Fitting linear mixed-effects models using lme4. arXiv preprint arXiv:1406.5823.

[bib4] Boehler C.N., Schevernels H., Hopf J.M., Stoppel C.M., Krebs R.M. (2014). Reward prospect rapidly speeds up response inhibition via reactive control. Cogn., Affect., Behav. Neurosci..

[bib5] Bolenz F., Eppinger B. (2022). Valence bias in metacontrol of decision making in adolescents and young adults. Child Dev..

[bib6] Bonnelle V., Manohar S., Behrens T., Husain M. (2016). Individual differences in premotor brain systems underlie behavioral apathy. Cereb. cortex.

[bib7] Botvinick M., Braver T. (2015). Motivation and cognitive control: from behavior to neural mechanism. Annu. Rev. Psychol..

[bib8] Braams B.R., van Duijvenvoorde A.C., Peper J.S., Crone E.A. (2015). Longitudinal changes in adolescent risk-taking: a comprehensive study of neural responses to rewards, pubertal development, and risk-taking behavior. J. Neurosci..

[bib9] Cao Z., Bennett M., Orr C., Icke I., Banaschewski T., Barker G.J. (2019). Mapping adolescent reward anticipation, receipt, and prediction error during the monetary incentive delay task. Human brain mapping.

[bib10] Chevalier N. (2018). Willing to think hard? The subjective value of cognitive effort in children. Child Dev..

[bib11] Christakou A., Gershman S.J., Niv Y., Simmons A., Brammer M., Rubia K. (2013). Neural and psychological maturation of decision-making in adolescence and young adulthood. J. Cogn. Neurosci..

[bib12] Collins A.G., Ciullo B., Frank M.J., Badre D. (2017). Working memory load strengthens reward prediction errors. J. Neurosci..

[bib13] Collins A.G., Frank M.J. (2012). How much of reinforcement learning is working memory, not reinforcement learning? A behavioral, computational, and neurogenetic analysis. Eur. J. Neurosci..

[bib14] Collins A.G., Frank M.J. (2013). Cognitive control over learning: creating, clustering, and generalizing task-set structure. Psychol. Rev..

[bib15] Crone E.A., Dahl R.E. (2012). Understanding adolescence as a period of social– affective engagement and goal flexibility. Nat. Rev. Neurosci..

[bib16] Crone E.A., Steinbeis N. (2017). Neural perspectives on cognitive control development during childhood and adolescence. Trends Cogn. Sci..

[bib17] Davidow J.Y., Foerde K., Galván A., Shohamy D. (2016). An upside to reward sensitivity: the hippocampus supports enhanced reinforcement learning in adolescence. Neuron.

[bib18] Davidow J.Y., Insel C., Somerville L.H. (2018). Adolescent development of value-guided goal pursuit. Trends Cogn. Sci..

[bib19] Deci E.L. (1971). Effects of externally mediated rewards on intrinsic motivation. J. Personal. Soc. Psychol..

[bib20] Eckstein, M.K., Master, S.L., Dahl, R.E., Wilbrecht, L., & Collins, A.G.E. (2021). The unique advantage of adolescents in probabilistic reversal: Reinforcement learning and Bayesian inference provide adequate and complementary models. 10.1016/j.dcn.2022.101106 PMC9108470 35537273

[bib21] Fouragnan E., Retzler C., Philiastides M.G. (2018). Separate neural representations of prediction error valence and surprise: Evidence from an fMRI meta-analysis. Hum. brain Mapp..

[bib22] Fulmer S.M., Frijters J.C. (2009). A review of self-report and alternative approaches in the measurement of student motivation. Educ. Psychol. Rev..

[bib23] Hauser T.U., Iannaccone R., Walitza S., Brandeis D., Brem S. (2015). Cognitive flexibility in adolescence: Neural and behavioral mechanisms of reward prediction error processing in adaptive decision making during development. NeuroImage.

[bib24] Holleman G.A., Hooge I.T., Kemner C., Hessels R.S. (2020). The ‘real-world approach’and its problems: A critique of the term ecological validity. Front. Psychol..

[bib25] Holroyd C.B., Yeung N. (2012). Motivation of extended behaviors by anterior cingulate cortex. Trends Cogn. Sci..

[bib26] Insel C., Charifson M., Somerville L.H. (2019). Neurodevelopmental shifts in learned value transfer on cognitive control during adolescence. Dev. Cogn. Neurosci..

[bib27] Insel C., Kastman E.K., Glenn C.R., Somerville L.H. (2017). Development of corticostriatal connectivity constrains goal-directed behavior during adolescence. Nat. Commun..

[bib28] Inzlicht M., Shenhav A., Olivola C.Y. (2018). The effort paradox: Effort is both costly and valued. Trends Cogn. Sci..

[bib29] Jarvis H., Stevenson I., Huynh A.Q., Babbage E., Coxon J., Chong T.T.J. (2022). Effort reinforces learning. J. Neurosci..

[bib30] Jimura K., Locke H.S., Braver T.S. (2010). Prefrontal cortex mediation of cognitive enhancement in rewarding motivational contexts. Proc. Natl. Acad. Sci..

[bib31] Kool W., Gershman S.J., Cushman F.A. (2017). Cost-benefit arbitration between multiple reinforcement-learning systems. Psychol. Sci..

[bib32] Kool W., Shenhav A., Botvinick M.M. (2017). Cognitive control as cost-benefit decision making. Wiley Handb. Cogn. Control.

[bib33] Kramer A.W., Huizenga H.M., Van Duijvenvoorde, Krabbendam L. (2024). Do I want to learn today? Day-to-day variations in adolescents’ academic motivation and effort. Learning and Motivation.

[bib34] Kramer A.W., Schaaf J.V., Huizenga H.M. (2023). How much do you want to learn? High-school students' willingness to invest effort in valenced feedback- learning tasks. Learn. Individ. Differ..

[bib35] Kramer A.W., Van Duijvenvoorde A.C., Krabbendam L., Huizenga H.M. (2021). Individual differences in adolescents’ willingness to invest cognitive effort: Relation to need for cognition, motivation and cognitive capacity. Cogn. Dev..

[bib36] Krebs R.M., Boehler C.N., Egner T., Woldorff M.G. (2011). The neural underpinnings of how reward associations can both guide and misguide attention. J. Neurosci..

[bib37] Kurzban R. (2016). The sense of effort. Curr. Opin. Psychol..

[bib38] Leotti L.A., Wager T.D. (2010). Motivational influences on response inhibition measures. J. Exp. Psychol.: Hum. Percept. Perform..

[bib39] Liu Y., Yang Y., Bai X., Chen Y., Mo L. (2022). Do immediate external rewards really enhance intrinsic motivation?. Front. Psychol..

[bib40] Locke H.S., Braver T.S. (2008). Motivational influences on cognitive control: behavior, brain activation, and individual differences. Cogn., Affect., Behav. Neurosci..

[bib41] Luna B. (2009). Developmental changes in cognitive control through adolescence. Adv. Child Dev. Behav..

[bib42] Master S.L., Eckstein M.K., Gotlieb N., Dahl R., Wilbrecht L., Collins A.G. (2020). Disentangling the systems contributing to changes in learning during adolescence. Dev. Cogn. Neurosci..

[bib43] Miller E.K., Cohen J.D. (2001). An integrative theory of prefrontal cortex function. Annu. Rev. Neurosci..

[bib44] Nagase A.M., Onoda K., Foo J.C., Haji T., Akaishi R., Yamaguchi S., Morita K. (2018). Neural mechanisms for adaptive learned avoidance of mental effort. J. Neurosci..

[bib45] Padmala S., Pessoa L. (2011). Reward reduces conflict by enhancing attentional control and biasing visual cortical processing. J. Cogn. Neurosci..

[bib46] R Core Team. (2023). R: A language and environment for statistical computing. R Foundation for Statistical Computing, Vienna, Austria. https://www.R- project.org/.

[bib47] Satterthwaite T.D., Wolf D.H., Erus G., Ruparel K., Elliott M.A., Gennatas E.D., Gur R.E. (2013). Functional maturation of the executive system during adolescence. J. Neurosci..

[bib48] Schaaf J., Miletic S., Duijvenvoorde A.C.K. van, Huizenga H.M. (2024). Interpretation of individual differences in computational neuroscience. Dev. Cogn. Neurosci..

[bib49] Schmidt L., Lebreton M., Cléry-Melin M.L., Daunizeau J., Pessiglione M. (2012). Neural mechanisms underlying motivation of mental versus physical effort. PLoS Biol..

[bib50] Scholl J., Kolling N., Nelissen N., Wittmann M.K., Harmer C.J., Rushworth M.F. (2015). The good, the bad, and the irrelevant: neural mechanisms of learning real and hypothetical rewards and effort. J. Neurosci..

[bib51] Scholl J., Kolling N., Nelissen N., Stagg C.J., Harmer C.J., Rushworth M.F. (2017). Excitation and inhibition in anterior cingulate predict use of past experiences. Elife.

[bib52] Schreuders E., Braams B.R., Blankenstein N.E., Peper J.S., Güroğlu B., Crone E.A. (2018). Contributions of reward sensitivity to ventral striatum activity across adolescence and early adulthood. Child Dev..

[bib53] Schultz W. (2016). Dopamine reward prediction error coding. Dialog-. Clin. Neurosci..

[bib54] Schultz W. (2017). Reward prediction error. Curr. Biol..

[bib55] Seaman K.L., Gorlick M.A., Vekaria K.M., Hsu M., Zald D.H., Samanez-Larkin G.R. (2016). Adult age differences in decision making across domains: Increased discounting of social and health-related rewards. Psychol. Aging.

[bib56] Shenhav A., Botvinick M.M., Cohen J.D. (2013). The expected value of control: an integrative theory of anterior cingulate cortex function. Neuron.

[bib57] Shenhav A., Cohen J.D., Botvinick M.M. (2016). Dorsal anterior cingulate cortex and the value of control. Nat. Neurosci..

[bib58] Shenhav A., Musslick S., Lieder F., Kool W., Griffiths T.L., Cohen J.D., Botvinick M.M. (2017). Toward a rational and mechanistic account of mental effort. Annu. Rev. Neurosci..

[bib59] Silverman M.H., Jedd K., Luciana M. (2015). Neural networks involved in adolescent reward processing: an activation likelihood estimation meta- analysis of functional neuroimaging studies. NeuroImage.

[bib60] Sutton, R.S., & Barto, A.G. (2018). Reinforcement learning: An introduction. MIT press.

[bib61] Telzer E.H. (2016). Dopaminergic reward sensitivity can promote adolescent health: A new perspective on the mechanism of ventral striatum activation. Dev. Cogn. Neurosci..

[bib62] Touroutoglou A., Andreano J., Dickerson B.C., Barrett L.F. (2020). The tenacious brain: How the anterior mid-cingulate contributes to achieving goals. Cortex.

[bib63] Van der Schaaf M.E., Warmerdam E., Crone E.A., Cools R. (2011). Distinct linear and non-linear trajectories of reward and punishment reversal learning during development: relevance for dopamine's role in adolescent decision making. Dev. Cogn. Neurosci..

[bib64] Van Duijvenvoorde A.C., Peters S., Braams B.R., Crone E.A. (2016). What motivates adolescents? Neural responses to rewards and their influence on adolescents’ risk taking, learning, and cognitive control. Neurosci. Biobehav. Rev..

[bib65] Vassena E., Holroyd C.B., Alexander W.H. (2017). Computational models of anterior cingulate cortex: At the crossroads between prediction and effort. Front. Neurosci..

[bib66] Verburg M., Snellings P., Zeguers M.H.T., Huizenga H.M. (2019). Positive-blank versus negative-blank feedback learning in children and adults. Quarterly journal of experimental psychology.

[bib67] Vlasceanu M., Doell K.C., Bak-Coleman J.B., Todorova B., Berkebile-Weinberg M.M., Grayson S.J., Lutz A.E. (2024). Addressing climate change with behavioral science: A global intervention tournament in 63 countries. Sci. Adv..

[bib68] Wagner A.R., Rescorla R.A. (1972). Inhibition in Pavlovian conditioning: Application of a theory. Inhibition and learning.

[bib69] Waller L., Erk S., Pozzi E., Toenders Y.J., Haswell C.C., Büttner M., Veer I.M. (2022). ENIGMA HALFpipe: Interactive, reproducible, and efficient analysis for resting-state and task-based fMRI data. Hum. Brain Mapp..

[bib70] Westbrook A., Braver T.S. (2015). Cognitive effort: A neuroeconomic approach. Cogn., Affect., Behav. Neurosci..

[bib71] Westbrook A., Kester D., Braver T.S. (2013). What is the subjective cost of cognitive effort? Load, trait, and aging effects revealed by economic preference. PloS One.

[bib72] Westbrook A., Lamichhane B., Braver T. (2019). The subjective value of cognitive effort is encoded by a domain-general valuation network. J. Neurosci..

[bib73] Yarkoni T. (2022). The generalizability crisis. Behav. Brain Sci..

[bib74] Yee D.M., Braver T.S. (2018). Interactions of motivation and cognitive control. Curr. Opin. Behav. Sci..

[bib75] Yee D.M., Crawford J.L., Lamichhane B., Braver T.S. (2021). Dorsal anterior cingulate cortex encodes the integrated incentive motivational value of cognitive task performance. J. Neurosci..

[bib76] Yoo A.H., Collins A.G. (2022). How working memory and reinforcement learning are intertwined: A cognitive, neural, and computational perspective. J. Cogn. Neurosci..

[bib77] Zendehrouh S., Gharibzadeh S., Towhidkhah F. (2014). Reinforcement-conflict based control: An integrative model of error detection in anterior cingulate cortex. Neurocomputing.

