# Supplementary material for: Make it worth it: Effort-reward modulations on reinforcement-learning and prediction-error signaling across adolescence
Source: Dev Cogn Neurosci. 2025 Apr 15;73:101559. doi: 10.1016/j.dcn.2025.101559 (PMC12063155; doi:10.1016/j.dcn.2025.101559)
Supplement: Table S1 — Supplementary material [file mmc1.docx]

**Supplementary Online Materials (SOM)**

## Descriptives of behavioral measures

**Table S1**

*Descriptives of behavioral measures.*

| Variable | N | Mean | SD | Minimum | Maximum |
| --- | --- | --- | --- | --- | --- |
| 1.Subjective value of effort | 143 | 1.42 | 0.53 | 0.16 | 1.98 |
| 2.Age | 146 | 19.71 | 3.28 | 13.23 | 25.98 |
| 3.Reward sensitivity (BAS) | 143 | 17.69 | 2.26 | 5.00 | 20.00 |
| 4.Performance | 146 | 85.36 | 9.97 | 48.06 | 98.05 |
| 5.Motivation ratings | 144 | 4.39 | 0.55 | 1.00 | 5.00 |
| 6.Effort ratings | 144 | 2.75 | 0.76 | 1.00 | 5.00 |

*Note*: Range is observed.

**Table S2***Average performance on the reinforcement learning task (in percentage correct) and average reaction times (in milliseconds) per condition with standard deviations between brackets.*

| **Condition** | **Accuracy** | **Reaction times** |
| --- | --- | --- |
| Low effort – low reward | 0.87 *(0.13)* | 955.56 *(143.01)* |
| Low effort – high reward | 0.89 *(0.11)* | 941.16 *(145.13)* |
| High effort – low reward | 0.80 *(0.16)* | 1001.32 *(151.53)* |
| High effort – high reward | 0.84 *(0.14)* | 1006.97 *(144.49)* |

**Table S3***Correlations between behavioral measures*

|  | 1 | 2 | 3 | 4 | 5 |
| --- | --- | --- | --- | --- | --- |
| 1.Subjective value of effort | - |  |  |  |  |
| 2.Age | -.08 | - |  |  |  |
| 3.Reward sensitivity (BAS) | .08 | -.03 |  |  |  |
| 4.Performance | .31*** | .10 | .18* |  |  |
| 5.Motivation ratings | .16 | -.04 | .12 | .09 |  |
| 6.Effort ratings | .22** | .04 | -.08 | -.40*** | -.05 |

Note: *p < .05, **p < .01, ***p < .001.

**Figure S1***Distribution of age.*

**
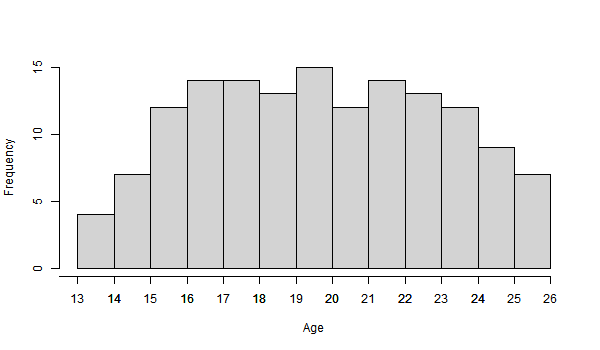
**

*Note*: N = 146.

## Computational modeling of behavioral data

We modeled choice accuracy using the Rescorla-Wagner reinforcement learning model (Rescorla & Wagner, 1972; Sutton & Barto, 2018). The model describes how people learn the value of stimuli (*Q*, in our case pseudo-word spellings) over trials (*t*) based on prediction errors (*PE*), that is, differences between observed rewards (*R*) and current stimulus values *Q*. To what extent prediction errors are used to update stimulus values is controlled by the learning rate parameter (*LR*):

$$Q\left( t+1 \right)=Q\left( t \right)+LR*PE\left( t \right)$$

with $PE\left( t \right)=R\left( t \right)-Q(t)$

The learning rate parameter varies between 0, indicating people do not use prediction errors to update stimulus values, and 1, indicating people update stimulus values to the last observed reward. In turn, stimulus values are translated into the probability of choosing each stimulus using the following choice function (Luce, 1959):

$$P(choose stimulus 1)=\frac{1}{1+e^{-InvT(Q_{1}-Q_{2})}}$$

The inverse temperature parameter (*InvT*) ranges from 0 to 50 (Gershman, 2016) and controls to what extent people use the difference between the two stimulus values (*Q_1_ – Q_2_*) to inform future choice. The lower this parameter, the less people use value differences, leading to more stochastic choice behavior.

In our extended model, we allow for main and interaction effects of age, effort condition and reward condition. Inspired by Bolenz and Eppinger (2022), we fitted our reinforcement learning models with and without an age effect in the model. In the non-developmental model we regressed learning rates and inverse temperatures on effort condition (low = -1, high = 1), reward condition (low = -1, high = 1), and their two-way interaction. In the developmental model, we regressed learning rate and inverse temperature parameters on effort condition, reward condition, linear age (mean-centered and scaled), and all two- and three-way interactions.

We implemented the reinforcement learning model in a hierarchical Bayesian framework, that is, in JAGS (Plummer, 2003) and performed estimation using the R2Jags package (Su & Yajima, 2015). We did so because hierarchical Bayesian estimation improves the reliability of individual parameter estimates (Katahira, 2016) and subsequently helps detect effects in model-based fMRI analyses (Ahn et al., 2011). Also, by estimating regression coefficients in a Bayesian framework, we obtained posterior distributions for each regression coefficient, quantifying uncertainty around the point estimates (i.e., the means of these distributions).

Specifically, we implemented normal distributions centered around zero with a large standard deviation as prior distributions on the population-level mean of all regression coefficients: $\beta\sim N(0, 10)$. For the population-level standard deviation, we used Cauchy distributions, truncated between 0 and 10. In JAGS, this is accomplished by specifying a Student *t* distribution with one degree of freedom: $sd(\beta) \sim{t(0, 5, 1)}_{I(0,10)}$. We subsequently drew participant-specific regression coefficients from the resulting population-level distributions, $\beta_{i} \sim N(\beta, sd(\beta))$, and then predicted participant-specific learning rates and inverse temperatures using these coefficients. Note that learning rates were transformed from a [-Inf, Inf] scale (as indicated by the normal distribution) to a [0, 1] scale by means of a probit transformation (Lee & Wagenmakers, 2013); inverse temperatures were transformed to a [0, 50] scale by first performing a probit transformation and then multiplying by 50. Modeling code is shared upon request.
 To assess whether the non-developmental or the developmental model fitted the data better, we compared the deviance information criterion (DIC; Spiegelhalter et al., 2002), a model fit index for complex hierarchical models. We interpreted results from the non-developmental model because this model fitted the data best (as indicated by a lower DIC value). To assess whether effort and reward affected learning rates and/or inverse temperatures, we investigated whether zero lay within the 95% highest-density interval (similar to a frequentist 95% confidence interval) of the posterior distributions of the regression coefficients. As shown in Figure S2, results only showed a marginal effect of effort on inverse temperatures (90% HDI: [-0.23; -0.009]), indicating, if anything, more stochastic choice behavior (i.e., lower inverse temperatures) in the high as compared to the low effort condition. There was no effect of reward nor an interaction effect between reward and effort.

From the non-developmental model, the estimated prediction errors (PE; positive and negative) and value estimates (Q) were subsequently used as trial-level parametric regressors in the fMRI analyses, modulating respectively the moment of feedback onset (positive prediction error, negative prediction error), and the moment of choice (value). The regressors were mean-centered per participant per regressor across conditions, they were not standardized.

**Figure S2***Estimates from best-fitting computational model of effects of effort, reward and their interaction on participants’ learning rates (left panels) and inverse temperatures (right panels), with 95% highest density intervals (black solid lines) and 90% highest density intervals (black dotted lines). Blue dashed line denotes 0.*
**
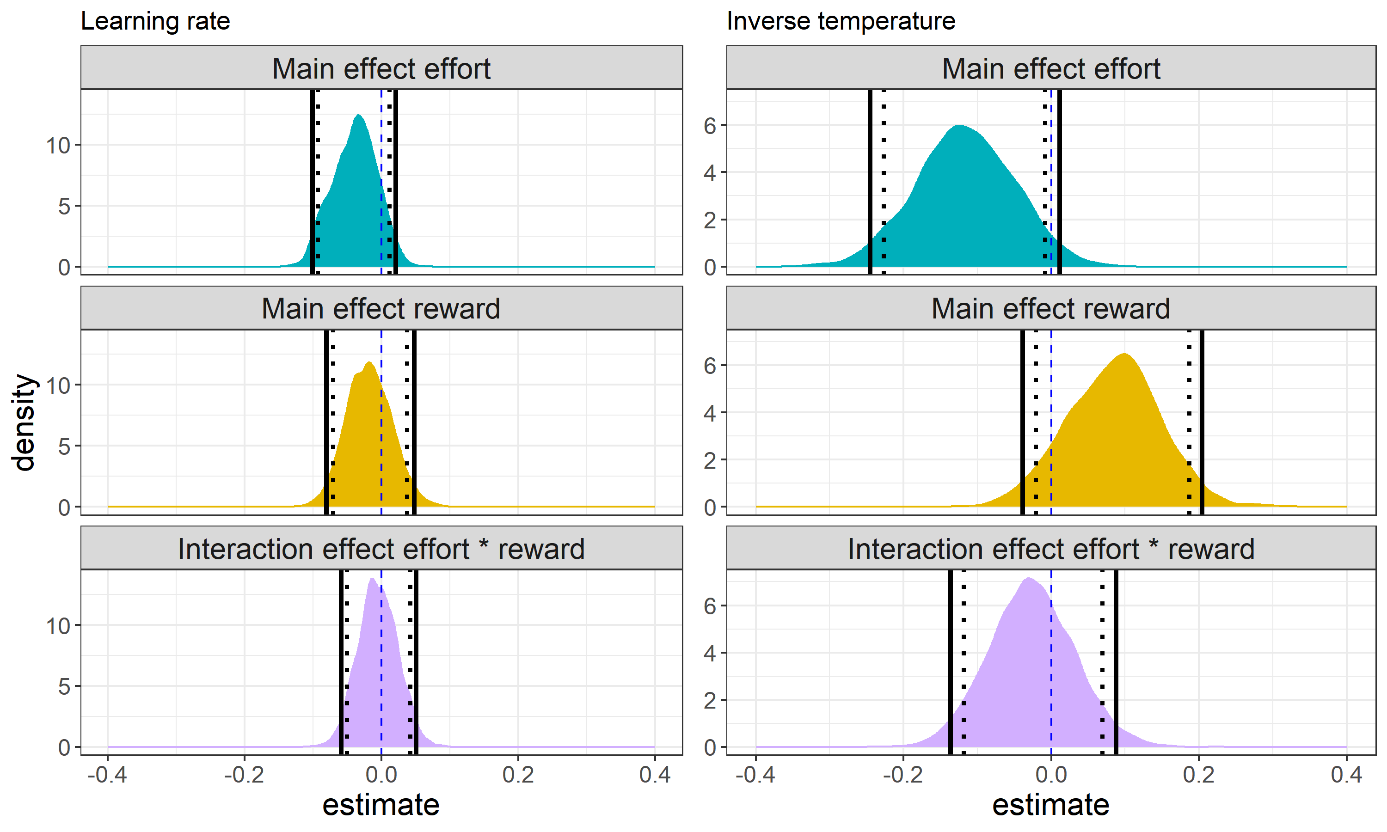
**

## Subjective ratings of effort and motivation

**Table S4***Hierarchical model fit on subjective ratings of effort and motivation for the reinforcement learning tasks*

| *Subjective ratings of effort* | | | | | |
| --- | --- | --- | --- | --- | --- |
| Predictors | *Β* | *SE* | 95% CI | *t* | *p* |
| **Intercept** | **2.75** | **0.06** | **2.62 – 2.88** | **43.09** | **< .001** |
| **effort** | **0.47** | **0.04** | **0.41 – 0.53** | **11.36** | **< .001** |
| reward | -0.05 | 0.02 | -0.10 – 0.01 | -2.30 | .117 |
| age | 0.03 | 0.06 | -0.10 – 0.15 | 0.42 | .672 |
| effort * reward | -0.01 | 0.02 | -0.05 – 0.02 | -0.71 | .478 |
| effort * age | 0.03 | 0.04 | -0.05 – 0.11 | 0.74 | .462 |
| **reward * age** | **0.06** | **0.02** | **0.02 – 0.10** | **3.03** | **.039** |
| effort * reward * age | 0.00 | 0.02 | -0.04 – 0.04 | 0.03 | .980 |
| Random effects | | | | | |
| σ^2^ | 0.47 |  |  |  |  |
| Random intercept | 0.47 |  |  |  |  |
| ICC | 0.50 |  |  |  |  |
| Marginal R^2^ / Conditional R^2^ | 0.20 / 0.59 |  |  |  |  |
| N / N_observations_ | 144 / 576 |  |  |  |  |
| *Subjective ratings of motivation* | | | | | |
| Predictors | *Β* | *SE* | 95% CI | *t* | *p* |
| **Intercept** | **4.39** | **0.05** | **4.30 – 4.48** | **95.43** | **< .001** |
| effort | 0.01 | 0.02 | -0.03 – 0.05 | 0.36 | .716 |
| **reward** | **0.26** | **0.02** | **0.22 – 0.30** | **13.66** | **< .001** |
| age | -0.02 | 0.05 | -0.11 – 0.07 | -0.50 | .615 |
| effort * reward | -0.01 | 0.02 | -0.05 – 0.03 | -0.43 | .683 |
| effort * age | 0.01 | 0.02 | -0.03 – 0.06 | 0.62 | .493 |
| **reward * age** | **-0.05** | **0.02** | **-0.09 - -0.01** | **-2.47** | **.019** |
| effort * reward * age | 0.01 | 0.02 | -0.03 – 0.05 | 0.74 | .483 |
| Random effects | | | | | |
| σ^2^ | 0.23 |  |  |  |  |
| Random intercept | 0.25 |  |  |  |  |
| ICC | 0.52 |  |  |  |  |
| Marginal R^2^ / Conditional R^2^ | 0.13 / 0.58 |  |  |  |  |
| N / N_observations_ | 144 / 576 |  |  |  |  |

**Figure S3***Subjective ratings of effort (top panels) and motivation (bottom panels) across effort and reward conditions (left panels) and across age (right panels).*

**
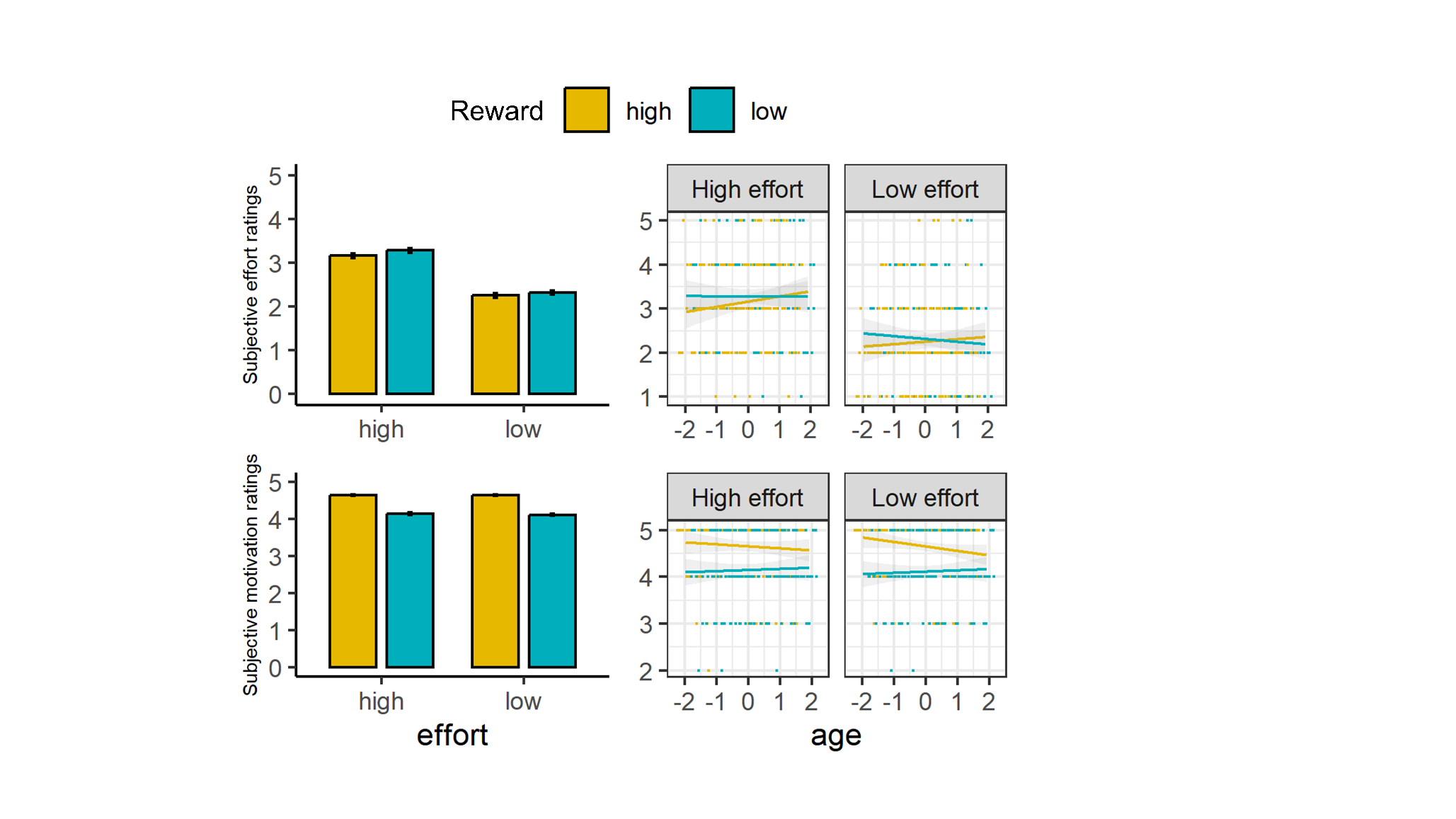
**

## Results with age (Hypotheses 1 and 2)

**Table S5***Model fit on learning accuracy in the reinforcement learning task*

| A*ccuracy* | | | | | |  |
| --- | --- | --- | --- | --- | --- | --- |
| Predictor | *Β* | *SE* | 95% CI | z | *p* |  |
| **Intercept** | **2.05** | **0.08** | **1.90 – 2.20** | **27.25** | **< .001** |  |
| **trial** | **2.78** | **0.10** | **2.60 – 2.97** | **29.28** | **< .001** |  |
| **effort** | **-0.27** | **0.02** | **-0.31 – -0.22** | **-12.40** | **< .001** |  |
| **reward** | **0.14** | **0.02** | **0.09 – 0.18** | **6.12** | **< .001** |  |
| age | 0.11 | 0.08 | -0.04 – 0.26 | 1.48 | .138 |  |
| **effort * trial** | **-0.38** | **0.09** | **-0.57 – -0.20** | **-4.09** | **< .001** |  |
| **trial * reward** | **0.18** | **0.09** | **-0.00 – 0.37** | **1.97** | **.051** |  |
| effort * reward | -0.01 | 0.02 | -0.05 – 0.03 | -0.66 | .580 |  |
| **trial * age** | **0.25** | **0.09** | **0.07 – 0.44** | **2.69** | **.007** |  |
| **effort * age** | **-0.07** | **0.02** | **-0.11 – -0.03** | **-3.20** | **.001** |  |
| **reward * age** | **-0.07** | **0.02** | **-0.12 – -0.03** | **-3.44** | **.001** |  |
| effort * trial * reward | -0.07 | 0.09 | -0.25 – 0.12 | -0.67 | .477 |  |
| effort * trial * age | -0.13 | 0.09 | -0.32 – 0.05 | -1.44 | .150 |  |
| trial * reward * age | 0.06 | 0.09 | -0.12 – 0.24 | 0.63 | .528 |  |
| **effort * reward * age** | **-0.04** | **0.02** | **-0.09 – -0.00** | **-2.04** | **.041** |  |
| effort * reward * age * trial | 0.06 | 0.09 | -0.12 – 0.24 | 0.65 | .516 |  |
| Random effects | | | | | |  |
| σ^2^ | 3.29 |  |  |  |  |  |
| Random intercept | 0.74 |  |  |  |  |  |
| ICC | 0.18 |  |  |  |  |  |
| Marginal R^2^ / Conditional R^2^ | 0.11 / 0.28 |  |  |  |  |  |
| N / N_observations_ | 146 / 26280 | |  |  |  |  |
| *Accuracy in low effort tasks* | | | | | |  |
| Predictor | *Β* | *SE* | 95% CI | z | *p* | *p-bonf** |
| **Intercept** | **2.33** | **0.08** | **2.16 – 2.49** | **27.56** | **< .001** | **< .001** |
| **trial** | **3,23** | **0.17** | **2.90 – 3.55** | **19.48** | **< .001** | **< .001** |
| **reward** | **0.15** | **0.04** | **0.08 – 0.23** | **4.06** | **< .001** | **< .001** |
| **age** | **0.17** | **0.08** | **0.00 – 0.33** | **2.01** | **.045** | .090 |
| reward * trial | 0.26 | 0.16 | -0.06 – 0.58 | 1.56 | .118 | .236 |
| **trial * age** | **0.39** | **0.17** | **0.06 – 0.71** | **2.34** | **.019** | **.038** |
| reward * age | -0.03 | 0.04 | -0.11 – -0.04 | -0.85 | .395 | .790 |
| reward * trial * age | -0.00 | 0.16 | -0.32 – 0.32 | -0.02 | .984 | 1 |
| Random effects | | | | | |  |
| σ^2^ | 3.29 |  |  |  |  |  |
| Random intercept | 0.76 |  |  |  |  |  |
| ICC | 0.19 |  |  |  |  |  |
| Marginal R^2^ / Conditional R^2^ | 0.14 / 0.30 |  |  |  |  |  |
| N / N_observations_ | 146 / 8760 | |  |  |  |  |
| *Accuracy in high effort tasks* | | | | | |  |
| Predictor | *Β* | *SE* | 95% CI | z | *p* | *p-bonf** |
| **Intercept** | **1.83** | **0.08** | **1.67 – 1.99** | **22.03** | **< .001** | **< .001** |
| **trial** | **2.46** | **0.10** | **2.27 – 2.65** | **25.38** | **< .001** | **< .001** |
| **reward** | **0.13** | **0.02** | **0.08 – 0.17** | **5.82** | **< .001** | **< .001** |
| age | 0.04 | 0.08 | -0.12 – 0.21 | 0.54 | .593 | 1 |
| reward * trial | 0.12 | 0.10 | -0.07 – 0.30 | 1.22 | .223 | .446 |
| trial * age | 0.13 | 0.10 | 0.06 – 0.32 | 1.36 | .175 | .350 |
| **reward * age** | **-0.12** | **0.02** | **-0.16 – 0.08** | **-5.51** | **< .001** | **< .001** |
| reward * trial * age | 0.13 | 0.09 | -0.06 – 0.31 | 1.34 | .180 | .360 |
| Random effects | | | | | |  |
| σ^2^ | 3.29 |  |  |  |  |  |
| Random intercept | 0.91 |  |  |  |  |  |
| ICC | 0.22 |  |  |  |  |  |
| Marginal R^2^ / Conditional R^2^ | 0.08 / 0.28 |  |  |  |  |  |
| N / N_observations_ | 146 / 17520 |  |  |  |  |  |

## *Note*: *p-values have been Bonferroni corrected (multiplied by 2).

**Table S6***Model fit on learning accuracy in the reinforcement learning task including covariates IQ and sex*

| A*ccuracy* | | | | | |  |
| --- | --- | --- | --- | --- | --- | --- |
| Predictor | *Β* | *SE* | 95% CI | z | *p* |  |
| **Intercept** | **2.05** | **0.07** | **1.90 – 2.20** | **28.89** | **< .001** |  |
| **trial** | **2.87** | **0.10** | **2.60 – 3.06** | **29.27** | **< .001** |  |
| **effort** | **-0.28** | **0.02** | **-0.32 – -0.23** | **-12.40** | **< .001** |  |
| **reward** | **0.13** | **0.02** | **0.09 – 0.18** | **5.90** | **< .001** |  |
| age | 0.14 | 0.07 | -0.00 – 0.29 | 1.92 | .055 |  |
| **IQ** | **0.27** | **0.07** | **0.12 – 0.41** | **3.66** | **< .001** |  |
| sex | 0.06 | 0.07 | -0.08 – 0.20 | 0.82 | .415 |  |
| **trial * reward** | **0.22** | **0.10** | **0.03 – 0.41** | **2.24** | **.025** |  |
| trial * age | 0.13 | 0.10 | -0.07 – 0.32 | 1.29 | .198 |  |
| **reward * age** | **-0.07** | **0.02** | **-0.11 – -0.03** | **-3.20** | **.001** |  |
| **trial * effort** | **-0.41** | **0.10** | **-0.60 – -0.22** | **-4.21** | **<.001** |  |
| reward * effort | -0.01 | 0.02 | -0.06 – 0.03 | -0.52 | .601 |  |
| **age * effort** | **-0.07** | **0.02** | **-0.11 – 0.03** | **-3.23** | **.001** |  |
| trial * reward * age | 0.05 | 0.10 | -0.14 – 0.24 | 0.48 | .633 |  |
| trial * reward * effort | -0.04 | 0.10 | -0.23 – 0.15 | -0.38 | .705 |  |
| trial * age * effort | -0.11 | 0.10 | -0.30 – -0.08 | -1.13 | .258 |  |
| **reward * age * effort** | **-0.05** | **0.02** | **-0.10 - -0.01** | **-2.48** | **.013** |  |
| trial * reward * age * effort | 0.05 | 0.10 | -0.14 – 0.24 | 0.56 | .578 |  |
| Random effects | | | | | |  |
| σ^2^ | 3.29 |  |  |  |  |  |
| Random intercept | 0.63 |  |  |  |  |  |
| ICC | 0.16 |  |  |  |  |  |
| Marginal R^2^ / Conditional R^2^ | 0.13 / 0.28 |  |  |  |  |  |
| N / N_observations_ | 143 / 25380 | |  |  |  |  |

**Table S7***Results from RM ANOVAs on positive PE coding in ROIs*

| **ROI** | **Effect** | **DF** | ***F*** | ***p*** |
| --- | --- | --- | --- | --- |
| dACC | **Reward** | **(1, 576)** | **13.23** | **.03** |
|  | Age | (1, 576) | 2.39 | .12 |
|  | Effort | (1, 576) | 1.37 | .24 |
|  | Reward * age | (1, 576) | 0.53 | .47 |
|  | **Reward * effort** | **(1, 576)** | **3.97** | **.04** |
|  | Age * effort | (1, 576) | 0.32 | .57 |
|  | **Reward * age * effort** | (1, 576) | **4.77** | **.02** |
| Left Str | Reward | (1, 576) | 0.04 | .83 |
|  | Age | (1, 576) | 0.84 | .36 |
|  | Effort | (1, 576) | 0.00 | .95 |
|  | Reward * age | (1, 576) | 0.19 | .66 |
|  | Reward * effort | (1, 576) | 0.84 | .36 |
|  | **Age * effort** | **(1, 576)** | **4.54** | **.03** |
|  | Reward * age * effort | (1, 576) | 3.58 | .06 |
| Right Str | Reward | (1, 576) | 2.99 | .85 |
|  | **Age** | **(1, 576)** | **4.07** | **.04** |
|  | Effort | (1, 576) | 0.61 | .44 |
|  | Reward * age | (1, 576) | 0.09 | .77 |
|  | Reward * effort | (1, 576) | 1.91 | .17 |
|  | Age * effort | (1, 576) | 2.29 | .13 |
|  | Reward * age * effort | (1, 576) | 0.24 | .63 |
| vmPFC | **Reward** | **(1, 576)** | **7.24** | **<.001** |
|  | Age | (1, 576) | 0.26 | .61 |
|  | Effort | (1, 576) | 0.13 | .72 |
|  | Reward * age | (1, 576) | 0.02 | .90 |
|  | **Reward * effort** | **(1, 576)** | **8.14** | **<.001** |
|  | Age * effort | (1, 576) | 0.21 | .65 |
|  | Reward * age * effort | (1, 576) | 1.51 | .22 |

## Table S8

*Results from follow-up RM ANOVAs on positive PE coding in ROIs*

| **ROI** | **Effort level** | **Effect** | **DF** | ***F*** | ***p*** | ***p-bonf**** |
| --- | --- | --- | --- | --- | --- | --- |
| dACC | Low | Reward | (1, 288) | 1.19 | .28 | .56 |
|  | Low | Age | (1, 288) | 1.97 | .16 | .32 |
|  | Low | Reward * age | (1, 288) | 0.94 | .33 | .66 |
|  | **High** | **Reward** | **(1, 288)** | **18.3** | **<.001** | **<.001** |
|  | High | Age | (1, 288) | 0.55 | .46 | .92 |
|  | **High** | **Reward * age** | **(1, 288)** | **4.88** | **.03** | **.06** |
| Left Str | Low | Reward | (1, 288) | 0.58 | .45 | .90 |
|  | Low | Age | (1, 288) | 0.68 | .41 | .82 |
|  | Low | Reward * age | (1, 288) | 0.97 | .32 | .64 |
|  | High | Reward | (1, 288) | 0.28 | .60 | 1 |
|  | **High** | **Age** | **(1, 288)** | **5.12** | **.02** | **.04** |
|  | High | Reward * age | (1, 288) | 2.98 | .09 | .18 |
| Right Str | Low | Reward | (1, 288) | 0.05 | .82 | 1 |
|  | Low | Age | (1, 288) | 0.11 | .74 | 1 |
|  | Low | Reward * age | (1, 288) | 0.27 | .60 | 1 |
|  | **High** | **Reward** | **(1, 288)** | **5.53** | **.02** | **.04** |
|  | **High** | **Age** | **(1, 288)** | **7.12** | **.01** | **.02** |
|  | High | Reward * age | (1, 288) | 0.02 | .88 | 1 |
| vmPFC | Low | Reward | (1, 288) | 0.01 | .91 | 1 |
|  | Low | Age | (1, 288) | 0.45 | .50 | 1 |
|  | Low | Reward * age | (1, 288) | 0.88 | .35 | .70 |
|  | **High** | **Reward** | **(1, 288)** | **16.13** | **<.001** | **<.001** |
|  | High | Age | (1, 288) | 0.00 | .97 | 1 |
|  | High | Reward * age | (1, 288) | 0.64 | .43 | .86 |

## *Note*: *p-values have been Bonferroni corrected (multiplied by 2).

## Results with subjective value (SV) of effort (Hypothesis 3)

## Table S9 *Model fit on accuracy in the reinforcement learning task including SV as continuous predictor*

| *Accuracy* | | | | | |
| --- | --- | --- | --- | --- | --- |
| Predictor | *Β* | *SE* | 95% CI | z | *p* |
| **Intercept** | **2.04** | **0.07** | **1.90 – 2.17** | **29.82** | **< .001** |
| **reward** | **0.10** | **0.02** | **0.06 – 0.14** | **4.59** | **< .001** |
| **trial** | **2.68** | **0.08** | **2.51 – 2.84** | **31.89** | **< .001** |
| age | 0.06 | 0.07 | -0.07 – 0.19 | 0.87 | .38 |
| **effort** | **-0.21** | **0.02** | **-0.25 – -0.17** | **-10.16** | **< .001** |
| **SV** | **0.29** | **0.07** | **0.16 – 0.42** | **4.26** | **< .001** |
| reward * trial | 0.11 | 0.08 | -0.05 – 0.28 | 1.36 | .18 |
| **reward * age** | **-0.08** | **0.02** | **-0.12 – -0.05** | **-4.33** | **< .001** |
| trial * age | 0.07 | 0.08 | -0.10 – 0.23 | 0.80 | .42 |
| reward * effort | -0.01 | 0.02 | -0.05 – 0.03 | -0.36 | .72 |
| **effort * SV** | **0.18** | **0.02** | **0.13 – 0.22** | **8.52** | **< .001** |
| **reward * SV** | **-0.07** | **0.02** | **-0.11 - -0.03** | **-3.39** | **< .001** |
| trial * reward * age | 0.11 | 0.08 | -0.06 – 0.27 | 1.29 | .20 |
| reward * effort * SV | -0.02 | 0.02 | -0.15 – 0.12 | -0.88 | .84 |
| Random effects | | | | | |
| σ^2^ | 3.29 |  |  |  |  |
| Random intercept | 0.59 |  |  |  |  |
| ICC | 0.15 |  |  |  |  |
| Marginal R^2^ / Conditional R^2^ | 0.14 / 0.27 |  |  |  |  |
| N / N_observations_ | 143 / 25740 | |  |  |  |

**Table S10***Model fit on accuracy in the reinforcement learning task in the low SV group per effort level*

| *Accuracy in low SV group in low effort tasks* | | | | | |  |
| --- | --- | --- | --- | --- | --- | --- |
| Predictor | *Β* | *SE* | 95% CI | z | *p* | *p-bonf** |
| **Intercept** | **2.24** | **0.11** | **2.02 – 2.45** | **20.78** | **< .001** | **<.001** |
| **reward** | **0.14** | **0.05** | **0.03 – 0.24** | **2.58** | **.01** | **.04** |
| **trial** | **3.31** | **0.23** | **2.85 – 3.76** | **14.28** | **< .001** | **<.001** |
| age | 0.03 | 0.03 | -0.03 – 0.10 | 1.05 | .29 | 1 |
| reward * trial | -0.10 | 0.23 | -0.54 – 0.35 | -0.42 | .68 | 1 |
| reward * age | -0.01 | 0.02 | -0.04 – 0.02 | -0.46 | .65 | 1 |
| trial * age | 0.02 | 0.07 | -0.11 – 0.16 | 0.34 | .74 | 1 |
| reward * trial * age | -0.01 | 0.07 | -0.15 – 0.12 | -0.20 | .84 | 1 |
| Random effects | | | | | |  |
| σ^2^ | 3.29 |  |  |  |  |  |
| Random intercept | 0.58 |  |  |  |  |  |
| ICC | 0.15 |  |  |  |  |  |
| Marginal R^2^ / Conditional R^2^ | 0.14 / 0.27 |  |  |  |  |  |
| N / N_observations_ | 72 / 4320 | |  |  |  |  |
| *Accuracy in low SV group in high effort tasks* | | | | | |  |
| Predictor | *Β* | *SE* | 95% CI | z | *p* | *p-bonf** |
| **Intercept** | **1.40** | **0.10** | **1.22 – 1.59** | **14.57** | **< .001** | **< .001** |
| **reward** | **0.19** | **0.03** | **0.14 – 0.25** | **6.80** | **< .001** | **< .001** |
| **trial** | **2.35** | **0.12** | **2.11 – 2.59** | **18.93** | **< .001** | **< .001** |
| age | -0.00 | 0.03 | -0.06 – 0.06 | -0.06 | .95 | 1 |
| reward * trial | 0.17 | 0.12 | -0.07 – 0.41 | 1.39 | .16 | .64 |
| **reward * age** | **-0.04** | **0.01** | **-0.06 – 0.02** | **-4.86** | **<.001** | **< .001** |
| trial * age | -0.01 | 0.04 | -0.09– 0.06 | -0.36 | .72 | 1 |
| reward * trial * age | 0.02 | 0.04 | -0.05 – 0.09 | 0.52 | .60 | 1 |
| Random effects | | | | | |  |
| σ^2^ | 3.29 |  |  |  |  |  |
| Random intercept | 0.60 |  |  |  |  |  |
| ICC | 0.15 |  |  |  |  |  |
| Marginal R^2^ / Conditional R^2^ | 0.09 / 0.23 |  |  |  |  |  |
| N / N_observations_ | 72 / 8640 |  |  |  |  |  |

## *Note*: *p-values have been Bonferroni corrected (multiplied by 4).

**Table S11***Model fit on accuracy in the reinforcement learning task in the high SV group per effort level*

| *Accuracy in high SV group in low effort tasks* | | | | | |  |
| --- | --- | --- | --- | --- | --- | --- |
| Predictor | *Β* | *SE* | 95% CI | z | *p* | *p-bonf** |
| **Intercept** | **2.49** | **0.13** | **2.23 – 2.75** | **18.69** | **< .001** | **< .001** |
| **reward** | **0.12** | **0.06** | **0.01 – 0.23** | **2.14** | **.03** | **.12** |
| **trial** | **3.33** | **0.25** | **2.85 – 3.82** | **13.44** | **< .001** | **< .001** |
| age | 0.04 | 0.04 | -0.04 – 0.12 | 1.01 | .31 | 1 |
| **reward * trial** | **0.58** | **0.24** | **0.10 – 1.06** | **2.39** | **.02** | **.08** |
| reward * age | 0.01 | 0.02 | -0.02 – 0.05 | 0.82 | .41 | 1 |
| trial * age | 0.15 | 0.08 | -0.01 – 0.30 | 1.87 | .06 | .24 |
| reward * trial * age | 0.07 | 0.08 | -0.08 – 0.22 | 0.96 | .34 | 1 |
| Random effects | | | | | |  |
| σ^2^ | 3.29 |  |  |  |  |  |
| Random intercept | 0.95 |  |  |  |  |  |
| ICC | 0.22 |  |  |  |  |  |
| Marginal R^2^ / Conditional R^2^ | 0.14 / 0.33 |  |  |  |  |  |
| N / N_observations_ | 72 / 4320 | |  |  |  |  |
| *Accuracy in high SV group in high effort tasks* | | | | | |  |
| Predictor | *Β* | *SE* | 95% CI | z | *p* | *p-bonf** |
| **Intercept** | **2.28** | **0.12** | **2.05 – 2.52** | **19.42** | **< .001** | **< .001** |
| reward | -0.02 | 0.04 | -0.09 – 0.05 | -0.47 | .64 | 1 |
| **trial** | **2.80** | **0.16** | **2.48 – 3.11** | **17.41** | **< .001** | **< .001** |
| age | 0.02 | 0.04 | -0.05 – 0.10 | 0.61 | .54 | 1 |
| reward * trial | -0.04 | 0.16 | -0.36 – 0.27 | -0.28 | .78 | 1 |
| reward * age | -0.02 | 0.01 | -0.04 – 0.00 | -1.77 | .08 | .32 |
| trial * age | 0.09 | 0.05 | -0.01– 0.19 | 1.72 | .09 | .36 |
| reward * trial * age | 0.09 | 0.05 | -0.00 – 0.19 | 1.86 | .06 | .24 |
| Random effects | | | | | |  |
| σ^2^ | 3.29 |  |  |  |  |  |
| Random intercept | 0.86 |  |  |  |  |  |
| ICC | 0.21 |  |  |  |  |  |
| Marginal R^2^ / Conditional R^2^ | 0.10 / 0.28 |  |  |  |  |  |
| N / N_observations_ | 72 / 8640 |  |  |  |  |  |

## *Note*: *p-values have been Bonferroni corrected (multiplied by 4).

**Table S12***Results from RM ANOVAs on positive PE coding in ROIs per SV group*

| **ROI** | **SV group** | **Effort** | **Effect** | **DF** | ***F*** | ***p*** | ***p-bonf**** |
| --- | --- | --- | --- | --- | --- | --- | --- |
| dACC | **Low** | **Low** | **Reward** | **(1, 140)** | **5.11** | **.03** | .12 |
|  | Low | Low | Age | (1, 140) | 0.30 | .58 | 1 |
|  | Low | Low | Reward * age | (1, 140) | 0.03 | .87 | 1 |
|  | **Low** | **High** | **Reward** | **(1, 140)** | **11.35** | **<.001** | **<.001** |
|  | Low | High | Age | (1, 140) | 2.37 | .13 | .52 |
|  | **Low** | **High** | **Reward * age** | **(1, 140)** | **5.92** | **.02** | .08 |
|  | High | Low | Reward | (1, 140) | 0.32 | .57 | 1 |
|  | High | Low | Age | (1, 140) | 2.28 | .13 | .52 |
|  | High | Low | Reward * age | (1, 140) | 3.46 | .06 | .24 |
|  | **High** | **High** | **Reward** | **(1, 140)** | **8.20** | **<.001** | **<.001** |
|  | High | High | Age | (1, 140) | 0.75 | .39 | 1 |
|  | High | High | Reward * age | (1, 140) | 0.08 | .78 | 1 |
| Left Str | Low | Low | Reward | (1, 140) | 0.64 | .43 | 1 |
|  | Low | Low | Age | (1, 140) | 0.03 | .87 | 1 |
|  | Low | Low | Reward * age | (1, 140) | 0.02 | .88 | 1 |
|  | Low | High | Reward | (1, 140) | 1.84 | .18 | .72 |
|  | **Low** | **High** | **Age** | **(1, 140)** | **4.96** | **.03** | .12 |
|  | **Low** | **High** | **Reward * age** | **(1, 140)** | **5.60** | **.02** | .08 |
|  | High | Low | Reward | (1, 140) | 0.07 | .79 | 1 |
|  | High | Low | Age | (1, 140) | 0.79 | .38 | 1 |
|  | High | Low | Reward * age | (1, 140) | 2.64 | .11 | .44 |
|  | High | High | Reward | (1, 140) | 1.06 | .30 | 1 |
|  | High | High | Age | (1, 140) | 0.62 | .43 | 1 |
|  | High | High | Reward * age | (1, 140) | 0.05 | .82 | 1 |
| Right Str | Low | Low | Reward | (1, 140) | 0.68 | .41 | 1 |
|  | Low | Low | Age | (1, 140) | 0.00 | .97 | 1 |
|  | Low | Low | Reward * age | (1, 140) | 0.77 | .38 | 1 |
|  | Low | High | Reward | (1, 140) | 2.76 | .10 | .40 |
|  | **Low** | **High** | **Age** | **(1, 140)** | **5.43** | **.02** | **.08** |
|  | Low | High | Reward * age | (1, 140) | 1.60 | .21 | .84 |
|  | High | Low | Reward | (1, 140) | 0.10 | .75 | 1 |
|  | High | Low | Age | (1, 140) | 0.45 | .50 | 1 |
|  | High | Low | Reward * age | (1, 140) | 2.80 | .10 | .40 |
|  | High | High | Reward | (1, 140) | 2.95 | .09 | .36 |
|  | High | High | Age | (1, 140) | 1.95 | .16 | .64 |
|  | High | High | Reward * age | (1, 140) | 2.49 | .12 | .48 |
| vmPFC | Low | Low | Reward | (1, 140) | 2.4 | .12 | .48 |
|  | Low | Low | Age | (1, 140) | 0.46 | .50 | 1 |
|  | Low | Low | Reward * age | (1, 140) | 0.00 | .98 | 1 |
|  | **Low** | **High** | **Reward** | **(1, 140)** | **6.24** | **.01** | **.04** |
|  | Low | High | Age | (1, 140) | 0.39 | .53 | 1 |
|  | Low | High | Reward * age | (1, 140) | 0.49 | .48 | 1 |
|  | High | Low | Reward | (1, 140) | 2.13 | .15 | .60 |
|  | High | Low | Age | (1, 140) | 0.10 | .76 | 1 |
|  | High | Low | Reward * age | (1, 140) | 2.08 | .15 | .60 |
|  | **High** | **High** | **Reward** | **(1, 140)** | **7.75** | **.01** | **.04** |
|  | High | High | Age | (1, 140) | 0.24 | .63 | 1 |
|  | High | High | Reward * age | (1, 140) | 0.00 | .99 | 1 |

## *Note*: *p-values have been Bonferroni corrected (multiplied by 4).

## Whole brain results

**Table S13***Whole brain activations for positive prediction errors*

| Peak voxel | | | | | |
| --- | --- | --- | --- | --- | --- |
| Region | k (voxels) | x | y | z | Z-max |
| Main effect low>high effort (C1) |  |  |  |  |  |
| Superior frontal gyrus (L/R) | 965 | -2.5 | 39.5 | 39.5 | 4.41*** |
| Paracingulate gyrus (L/R) |  | 1.5 | 37.5 | 35.5 | 4.41*** |
| Anterior cingulate gyrus (R) |  | 9.5 | 47.5 | 1.5 | 3.86*** |
| Middle frontal gyrus (R) | 349 | 43.5 | 23.5 | 41.5 | 4.02*** |
| Lateral occipital cortex (superior) (R) | 204 | 37.5 | -64.5 | 45.4 | 3.76** |
| Supramarginal gyrus (posterior) (R) |  | 51.5 | -42.5 | 47.5 | 3.76** |
| Orbitofrontal cortex (R) | 196 | 39.5 | 17.5 | -16.5 | 3.82** |
| Frontal operculum cortex (R) |  | 33.5 | 23.5 | 5.5 | 3.82** |
| Middle temporal gyrus (posterior) (R) | 187 | 67.5 | -28.5 | -2.5 | 4.27* |
| *Positive age effect* |  |  |  |  |  |
| Inferior frontal gyrus (L) | 223 | -58.5 | 11.5 | 19.5 | 3.61** |
| Precentral gyrus (L) |  | -56.5 | 3.5 | 27.5 | 3.36** |
| Postcentral gyrus (L) |  | -54.5 | -10.5 | 29.5 | 3.13** |
| Precuneus cortex (L) | 154 | -2.5 | -58.5 | 55.5 | 3.56* |
| *Negative age effect* |  |  |  |  |  |
| No suprathreshold clusters |  |  |  |  |  |
| Main effect high>low effort (C2) |  |  |  |  |  |
| No suprathreshold clusters |  |  |  |  |  |
| *Positive age effect* |  |  |  |  |  |
| No suprathreshold clusters |  |  |  |  |  |
| *Negative age effect* |  |  |  |  |  |
| Mirrors positive age effect C1 |  |  |  |  |  |
| Main effect low>high reward (C4) |  |  |  |  |  |
| No suprathreshold voxels |  |  |  |  |  |
| *Positive age effect* |  |  |  |  |  |
| Cingulate gyrus (posterior) (L/R) | 425 | 1.5 | -32.5 | 37.5 | 4.15*** |
| Central opercular cortex (L) | 250 | -54.5 | -22.5 | 11.5 | 3.61** |
| Precentral gyrus (L) |  | -62.5 | 5.5 | 11.5 | 3.61** |
| Middle frontal gyrus (R) | 146 | 45.4 | 13.5 | 31.5 | 3.74* |
| *Negative age effect* |  |  |  |  |  |
| No suprathreshold voxels |  |  |  |  |  |
| Main effect high>low reward (C3) |  |  |  |  |  |
| Paracingulate gyrus (L/R) | 3017 | -0.5 | 53.5 | 3.5 | 4.46*** |
| Precentral gyrus (R) |  | 37.5 | 1.5 | 35.5 | 4.46*** |
| Frontal pole (R) |  | 21.5 | 45.5 | 37.5 | 4.25*** |
| Inferior frontal gyrus (R) |  | 53.5 | 25.5 | 7.5 | 4.25*** |
| Anterior cingulate cortex (L) |  | -8.5 | 29.5 | 17.5 | 4.18*** |
| Paracingulate gyrus (R) |  | 13.5 | 39.5 | 17.5 | 4.12*** |
| Cingulate gyrus (posterior) | 591 | -0.5 | -26.5 | 27.5 | 4.50*** |
| Cingulate gyrus (posterior) (L) |  | -0.5 | -20.5 | 43.5 | 4.50*** |
| Cingulate gyrus (posterior) (L) |  | -4.5 | -20.5 | 31.5 | 3.96*** |
| Cingulate gyrus (posterior) (R) |  | 1.5 | -26.5 | 27.5 | 3.96*** |
| Precuneus cortex (R) |  | 3.5 | -42.5 | 47.5 | 3.60*** |
| Middle temporal gyrus (posterior) (R) | 442 | 65.5 | -14.5 | -8.5 | 3.99*** |
| Superior temporal gyrus (R) |  | 45.5 | -28.5 | -0.5 | 3.93*** |
| Orbitofrontal cortex (R) | 298 | 47.5 | 19.5 | -10.5 | 4.32*** |
| Insular cortex (R) |  | 35.5 | 7.5 | -4.5 | 4.32*** |
| Supramarginal gyrus (posterior) (L) | 272 | -50.5 | -44.5 | 5.5 | 4.04*** |
| Planum temporale (L) |  | -50.5 | -32.5 | 9.5 | 3.69*** |
| Angular gyrus (R) | 272 | 51.5 | -48.5 | 25.5 | 4.21*** |
| Superior parietal lobe (R) | 225 | 37.5 | -54.5 | 55.5 | 3.73** |
| Supramarginal gyrus (posterior) (R) |  | 53.5 | -38.5 | 43.5 | 3.73** |
| *Positive age effect* |  |  |  |  |  |
| No suprathreshold voxels |  |  |  |  |  |
| *Negative age effect* |  |  |  |  |  |
| Mirrors positive age effect C4 |  |  |  |  |  |
| Low effort conditions: high>low reward (C14) |  |  |  |  |  |
| Middle frontal gyrus (L) | 165 | -36.5 | 23.5 | 35.5 | 3.71* |
| Middle frontal gyrus (L) |  | -44.5 | 21.5 | 33.5 | 3.26* |
| Middle frontal gyrus (L) |  | -36.5 | 23.5 | 35.5 | 3.17* |
| Middle frontal gyrus (L) |  | -36.5 | 23.5 | 375.5 | 3.09* |
| Caudate (L) |  | -18.5 | 10.5 | 25.5 | 2.90* |
| *Positive age effect* |  |  |  |  |  |
| No suprathreshold voxels |  |  |  |  |  |
| *Negative age effect* |  |  |  |  |  |
| No suprathreshold voxels |  |  |  |  |  |
| High effort conditions: high>low reward (C10) |  |  |  |  |  |
| Paracingulate gyrus (L/R) | 11190 | -0.5 | 53.5 | 1.5 | 5.22*** |
| Cingulate gyrus (posterior) (L/R) |  | -0.5 | -20.5 | 45.5 | 5.22*** |
| Frontal pole (R) |  | 15.5 | 47.5 | 37.5 | 5.14*** |
| Middle frontal gyrus (R) |  | 37.5 | 5.5 | 49.5 | 5.12*** |
| Cingulate gyrus (posterior) (L/R) |  | 1.5 | -28.5 | 45.5 | 5.11*** |
| Middle frontal gyrus (R) |  | 25.5 | 19.5 | 49.5 | 5.04*** |
| Angular gyrus (R) |  | 49.5 | -52.5 | 29.5 | 5.03*** |
| Orbitofrontal cortex (R) | 872 | 47.5 | 21.5 | -8.5 | 4.34*** |
| Orbitofrontal cortex (R) |  | 31.5 | 15.5 | -18.5 | 4.34** |
| Orbitofrontal cortex (R) |  | 45.5 | 19.5 | -2.5 | 3.85** |
| Angular gyrus (L) | 836 | -48.5 | -60.5 | 25.5 | 4.27*** |
| Planum temporale (L) |  | -50.5 | -36.5 | 9.5 | 4.27** |
| Lateral occipital cortex (L) |  | -36.5 | -70.5 | 25.5 | 4.07** |
| Orbitofrontal cortex (L) | 487 | -46.5 | 17.5 | -8.5 | 5.00*** |
| Insular cortex (L) |  | -34.5 | 15.5 | -10.5 | 5.00*** |
| Putamen (L) |  | -20.5 | 3.5 | -4.5 | 3.77*** |
| Superior frontal gyrus (L) | 371 | -26.5 | 31.5 | 47.5 | 5.98*** |
| Middle frontal gyrus (L) |  | -28.5 | 31.5 | 45.5 | 5.98*** |
| Frontal pole (L) |  | -22.5 | 45.5 | 37.5 | 4.48*** |
| Precentral gyrus (L) | 243 | -56.5 | -6.5 | 25.5 | 3.70** |
| Precentral gyrus (L) |  | -60.5 | -4.5 | 31.5 | 3.70** |
| Postcentral gyrus (L) |  | -62.5 | -4.5 | 27.5 | 3.32** |
| Superior frontal gyrus (L) | 212 | -24.5 | 5.5 | 59.5 | 4.30** |
| Precentral gyrus (L) |  | -30.5 | -16.5 | 59.5 | 3.34** |
| Precuneus cortex (L) | 177 | -6.5 | -78.5 | 35.5 | 3.76* |
| *Positive age effect* |  |  |  |  |  |
| No suprathreshold clusters |  |  |  |  |  |
| *Negative age effect* |  |  |  |  |  |
| Precuneus cortex (L/R) | 1915 | 1.5 | -56.5 | 25.5 | 4.43*** |
| Precuneus cortex (L/R) |  | 5.5 | -60.5 | 25.5 | 4.43*** |
| Lateral occipital cortex (R) |  | 35.5 | -58.5 | 49.5 | 4.24*** |
| Precuneus cortex (L) |  | -18.5 | -58.5 | 25.5 | 4.20*** |
| Lingual gyrus (L/R) | 1651 | -2.5 | -72.5 | -4.5 | 4.41*** |
| Superior temporal gyrus (posterior) (L) |  | -60.5 | -36.5 | 7.5 | 4.15*** |
| Planum temporale (L) |  | -60.5 | -12.5 | 5.5 | 4.06*** |
| Angular gyrus (L) |  | -46.5 | -52.5 | 19.5 | 4.03*** |
| Inferior temporal gyrus (L) |  | -52.5 | -56.5 | -8.5 | 3.93*** |
| Planum polare (R) | 582 | 43.5 | -12.5 | -4.5 | 4.42*** |
| Precentral gyrus (R) |  | 53.5 | -0.5 | 25.5 | 4.42*** |
| Postcentral gyrus (R) |  | 65.5 | -4.5 | 23.5 | 4.03*** |
| Superior temporal gyrus (posterior) (R) |  | 65.5 | -8.5 | 1.5 | 3.98*** |
| Insular cortex (R) |  | 35.5 | -12.5 | -0.5 | 3.60*** |
| Precentral gyrus (L) | 582 | -62.5 | 3.5 | 11.5 | 3.89*** |
| Postcentral gyrus (L) |  | -64.5 | -6.5 | 19.5 | 3.89*** |
| Central opercular cortex (L) |  | -54.5 | -12.5 | 17.5 | 3.71*** |
| Middle frontal gyrus (L) |  | -28.5 | 15.5 | 51.5 | 3.68*** |
| Precentral gyrus (L) |  | -52.5 | -0.5 | 35.5 | 3.60*** |
| Cingulate gyrus (anterior) (L/R) | 469 | -2.5 | 21.5 | 33.5 | 4.36*** |
| Cingulate gyrus (anterior) (L/R) |  | 1.5 | 15.5 | 35.5 | 4.36*** |
| Cingulate gyrus (anterior) (L/R) |  | -6.5 | 23.5 | 21.5 | 4.30*** |
| Paracingulate gyrus (L/R) |  | -0.5 | 33.5 | 31.5 | 3.73*** |
| Supramarginal gyrus (posterior) (R) | 437 | 57.5 | -34.5 | 43.5 | 4.41*** |
| Postcentral gyrus (R) |  | 41.5 | -22.5 | 53.5 | 3.75*** |
| Postcentral gyrus (R) |  | 51.5 | -16.5 | 43.5 | 3.36*** |
| Middle frontal gyrus (L) | 305 | -28.5 | 27.5 | 43.5 | 3.78*** |
| Frontal pole (L) |  | -20.5 | 51.5 | 19.5 | 3.78*** |
| Inferior frontal gyrus (R) | 238 | 53.5 | 9.5 | 19.5 | 3.97*** |
| Central opercular cortex (R) |  | 45.5 | -2.5 | 11.5 | 3.61*** |
| Lateral occipital cortex (superior) (R) | 211 | 43.5 | -72.5 | 21.5 | 4.48** |
| Caudate (R) | 211 | 17.5 | -12.5 | 25.5 | 3.58** |
| Middle frontal gyrus (R) |  | 35.5 | 7.5 | 35.5 | 3.58** |
| Frontal pole (R) | 199 | 23.5 | 41.5 | 35.5 | 4.38** |
| Supramarginal gyrus (anterior) (L) | 176 | -56.5 | -28.5 | 45.5 | 3.56* |
| Postcentral gyrus (L) |  | -52.5 | -22.5 | 39.5 | 3.56* |
| Precentral gyrus (L) | 170 | -32.5 | -6.5 | 47.5 | 3.74* |
| Heschl’s gyrus (R) | 160 | 37.5 | -22.5 | 3.5 | 3.59* |

*Note*: **p* < .05, ***p* < .01, ****p* < .001.

**Table S14***Whole brain activations for negative prediction errors*

| Peak voxel | | | | | |
| --- | --- | --- | --- | --- | --- |
| Region | k (voxels) | x | y | z | Z-max |
| Main effect low>high effort (C1) |  |  |  |  |  |
| Paracingulate gyrus | 174 | 1.5 | 47.5 | 15.5 | 3.39* |
| *Positive age effect* |  |  |  |  |  |
| Lingual gyrus (L) | 177 | -26.5 | -50.5 | 1.5 | 4.24* |
| Thalamus (L) |  | -14.5 | -34.5 | 9.5 | 2.70* |
| *Negative age effect* |  |  |  |  |  |
| No suprathreshold voxels |  |  |  |  |  |
| Main effect high>low effort (C2) |  |  |  |  |  |
| Temporal occipital fusiform cortex (R) | 351 | 23.5 | -56.5 | -12.5 | 4.31*** |
| Occipital fusiform gyrus (R) |  | 33.5 | -60.5 | -14.5 | 3.68*** |
| Temporal occipital fusiform cortex (L) | 316 | -22.5 | -58.5 | -14.5 | 3.79*** |
| Intracalcarine cortex (R) | 301 | 9.5 | -88.5 | 7.5 | 4.18*** |
| Lateral superior occipital cortex (L) | 249 | -28.5 | -74.5 | 23.5 | 3.71** |
| Lingual gyrus (L) | 214 | -8.5 | -78.5 | -8.5 | 3.78** |
| *Positive age effect* |  |  |  |  |  |
| No suprathreshold voxels |  |  |  |  |  |
| *Negative age effect* |  |  |  |  |  |
| Lingual gyrus (L) | 178 | -26.5 | -50.5 | 1.5 | 4.24* |
| Thalamus (L) |  | -14.5 | -34.5 | 9.5 | 2.70* |
| Main effect low>high reward (C4) |  |  |  |  |  |
| Caudate (L) | 172 | -12.5 | -2.5 | 15.5 | 3.90* |
| *Positive age effect* |  |  |  |  |  |
| Cingulate cortex (posterior) (R) | 429 | 11.5 | -50.5 | 29.5 | 4.21*** |
| Precuneus cortex (R) |  | 21.5 | -56.5 | 31.5 | 3.85** |
| Temporal pole (R) | 356 | 59.5 | 7.5 | -0.5 | 4.46*** |
| Superior temporal gyrus (R) |  | 65.5 | -26.5 | 1.5 | 3.52** |
| Planum temporale (L) | 243 | -48.5 | -30.5 | 11.5 | 4.12** |
| Superior temporal gyrus (L) |  | -56.5 | -30.5 | 5.5 | 3.97** |
| *Negative age effect* |  |  |  |  |  |
| No suprathreshold voxels |  |  |  |  |  |
| Main effect high>low reward (C3) |  |  |  |  |  |
| No suprathreshold voxels |  |  |  |  |  |
| *Positive age effect* |  |  |  |  |  |
| No suprathreshold voxels |  |  |  |  |  |
| *Negative age effect* |  |  |  |  |  |
| Cingulate gyrus (posterior) (R) | 429 | 11.5 | -50.5 | 29.5 | 5.10*** |
| Precuneus cortex (R) |  | 21.5 | -56.5 | 31.5 | 4.21*** |
| Temporal pole | 356 | 59.5 | 7.5 | -0.5 | 4.46*** |
| Planum temporale (L) | 245 | -48.5 | -30.5 | 11.5 | 4.12** |
| Superior temporal gyrus (L) |  | -62.5 | -24.5 | 3.5 | 3.30** |
| Low effort conditions: high>low reward (C14) |  |  |  |  |  |
| No suprathreshold voxels |  |  |  |  |  |
| *Positive age effect* |  |  |  |  |  |
| No suprathreshold voxels |  |  |  |  |  |
| *Negative age effect* |  |  |  |  |  |
| Angular gyrus (R) | 210 | 45.5 | -53.5 | 49.5 | 4.08** |
| Precuneus cortex (R) |  | 21.5 | -56.5 | 31.5 | 3.73** |
| High effort conditions: high>low reward (C10) |  |  |  |  |  |
| Precentral gyrus (L/R) | 5821 | -0.5 | -32.5 | 49.5 | 4.79*** |
| Precuneus cortex (L/R) |  | -8.5 | -62.5 | 49.5 | 4.79*** |
| Cingulate gyrus (posterior) (L/R) |  | -2.5 | -34.5 | 47.5 | 4.75*** |
| Superior temporal gyrus (posterior) (R) |  | 49.5 | -34.5 | 1.5 | 4.69*** |
| Paracingulate gyrus (L/R) | 846 | -0.5 | 51.5 | 19.5 | 4.09*** |
| Frontal pole (L) |  | -22.5 | 41.5 | 37.5 | 4.09*** |
| Superior frontal gyrus (L) |  | -26.5 | 25.5 | 53.5 | 3.96*** |
| Middle frontal gyrus (L) |  | -30.5 | 15.5 | 55.5 | 3.86*** |
| Lateral occipital cortex (superior) (L) | 582 | -46.5 | -64.5 | 25.5 | 4.04*** |
| Middle temporal gyrus (L) |  | -46.5 | -58.5 | 11.5 | 4.04*** |
| Frontal pole (L/R) | 384 | -0.5 | 57.5 | -2.5 | 3.97*** |
| Cingulate gyrus (anterior) (L) |  | -6.5 | 45.5 | 1.5 | 3.97*** |
| Paracingulate gyrus (R) |  | 5.5 | 53.5 | 7.5 | 3.85*** |
| Angular gyrus (R) | 299 | 43.5 | -58.5 | 19.5 | 4.55*** |
| Middle temporal gyrus (R) |  | 55.5 | -54.5 | 1.5 | 4.55*** |
| Lateral occipital cortex (R) |  | 41.5 | -60.5 | 21.5 | 4.30*** |
| *Positive age effect* |  |  |  |  |  |
| No suprathreshold voxels |  |  |  |  |  |
| *Negative age effect* |  |  |  |  |  |
| Heschl’s gyrus (L) | 1008 | -42.5 | -18.5 | 3.5 | 5.14*** |
| Planum temporale (L) |  | -48.5 | -30.5 | 7.5 | 5.14*** |
| Insular cortex (R) |  | 38.5 | -18.5 | 1.5 | 4.32*** |
| Supramarginal gyrus (L) |  | -62.5 | -48.5 | 11.5 | 4.25*** |
| Precuneus cortex (L/R) | 806 | -0.5 | -56.5 | 5.5 | 4.23*** |
| Cingulate cortex (posterior) (L) |  | -10.5 | -46.5 | 26.5 | 4.23*** |
| Precuneus cortex (R) |  | 13.5 | -54.5 | 51.5 | 3.96*** |
| Paracingulate gyrus (L/R) | 728 | -0.5 | 19.5 | 39.5 | 3.89*** |
| Paracingulate gyrus (L/R) |  | -0.5 | 51.5 | 9.5 | 3.89*** |
| Cingulate gyrus (anterior) (L) |  | -6.5 | 45.5 | 19.5 | 3.76*** |
| Paracingulate gyrus (L/R) |  | 1.5 | 21.5 | 39.5 | 3.62*** |
| Heschl’s gyrus (R) | 588 | 45.5 | -14.5 | -0.5 | 4.83*** |
| Supramarginal gyrus (R) |  | 45.5 | -34.5 | 7.5 | 4.83*** |
| Postcentral gyrus (R) |  | 65.5 | -4.5 | 11.5 | 3.61*** |
| Superior temporal gyrus (R) |  | 57.5 | -6.5 | -8.5 | 3.44*** |
| Cingulate cortex (posterior) (L/R) | 360 | -0.5 | -32.5 | 29.5 | 4.04*** |
| Cingulate gyrus (L) |  | -4.5 | -20.5 | 41.5 | 4.04*** |
| Angular gyrus (L) | 207 | -44.5 | -58.5 | 27.5 | 4.45** |
| Inferior frontal gyrus (L) | 173 | -54.5 | 15.5 | -2.5 | 4.28* |

*Note*: **p* < .05, ***p* < .01, ****p* < .001.

**Supplemental references**

Ahn, W.-Y., Krawitz, A., Kim, W., Busemeyer, J. R., & Brown, J. W. (2011). A model-based fMRI analysis with hierarchical Bayesian parameter estimation. *Journal of Neuroscience, Psychology, and Economics*, *4*(2), 95–110. <https://doi.org/10.1037/a0020684.A>

Gershman, S. J. (2016). Empirical priors for reinforcement learning models. *Journal of Mathematical Psychology*, *71*, 1–6. https://doi.org/10.1016/j.jmp.2016.01.006

Katahira, K. (2016). How hierarchical models improve point estimates of model parameters at the individual level. *Journal of Mathematical Psychology*, *73*, 37–58. https://doi.org/10.1016/j.jmp.2016.03.007

Lee, M. D., & Wagenmakers, E. J. (2013). *Bayesian cognitive modeling: A practical course*. Cambridge university press.

Luce, R. D. (1959). Individual Choice Behavior. In *Econometrica*. https://doi.org/10.2307/1911299

Plummer, M. (2003). JAGS: A program for analysis of Bayesian graphical models using Gibbs sampling. *Proceedings of the 3rd International Workshop on Distributed Statistical Computing*. https://doi.org/10.1.1.13.3406

Rescorla, R. A., & Wagner, A. R. (1972). A theory of Pavlovian conditioning: Variations in the effectiveness of reinforcement and nonreinforcement. In A. H. Black & W. F. Prokasy (Eds.), *Classical conditioning II: Current research and theory* (pp. 64–99). Appleton-Century-Crofts.

Spiegelhalter, D. J., Best, N. G., Carlin, B. P., & Van Der Linde, A. (2002). Bayesian measures of model complexity and fit. *Journal of the Royal Statistical Society. Series B: Statistical Methodology*, *64*(4), 583–616. https://doi.org/10.1111/1467-9868.00353

Su, Y.-S., & Yajima, M. (2015). R2jags: Using R to run “JAGS.” *R Packages*. https://doi.org/http://cran.r-project.org/package=R2jags

Sutton, R. S., & Barto, A. G. (2018). *Reinforcement Learning: An Introduction*. MIT Press. <https://doi.org/10.1016/S1364-6613(99)01331-5>
